# Analysing the protection from respiratory tract infections and allergic diseases early in life by human milk components: the PRIMA birth cohort

**DOI:** 10.1186/s12879-022-07107-w

**Published:** 2022-02-14

**Authors:** Arthur H. van Stigt, Katrien Oude Rengerink, Kitty W. M. Bloemenkamp, Wouter de Waal, Sabine M. P. J. Prevaes, Thuy-My Le, Femke van Wijk, Maaike Nederend, Anneke H. Hellinga, Christianne S. Lammers, Gerco den Hartog, Martijn J. C. van Herwijnen, Johan Garssen, Léon M. J. Knippels, Lilly M. Verhagen, Caroline G. M. de Theije, Alejandro Lopez-Rincon, Jeanette H. W. Leusen, Belinda van’t Land, Louis Bont, PRIMA Initiative Group, André C. Knulst, C. Kors van der Ent, Debbie van Baarle, Marca H. M. Wauben, Nynke Y. Rots, Elisabeth A. M. Sanders, Manon J. N. L. Benders, Laura A. M. P. Meulenbroek, Bernd Stahl, Aletta D. Kraneveld, Brigitte J. M. Buiteman, Tanja Voogt, Barbara van der Meij, Butsabong Lerkvaleekul, Eline Voogd, Christianne S. Lammers, Tariq A. Lalmahomed, Iris M. Brus, Daphne M. M. van Meerwijk, Sophie I. E. Jepma

**Affiliations:** 1 Center for Translational Immunology, University Medical Center Utrecht, Utrecht, The Netherlands; 2 Department of Biostatistics and Research Support, Clinical Trial Methodology, Julius Center for Health Sciences and Primary Care, University Medical Center Utrecht, Utrecht, The Netherlands; 3 Department of Gynaecology and Obstetrics, University Medical Center Utrecht, Utrecht, The Netherlands; 4 Department of Pediatrics, Diakonessenhuis, Utrecht, The Netherlands; 5 Department of Pediatric Pulmonology and Allergology, Wilhelmina Children’s Hospital/University Medical Center, Utrecht University, Utrecht, The Netherlands; 6 Department of Dermatology/Allergology, University Medical Center Utrecht, University of Utrecht, Utrecht, The Netherlands; 7 Centre for Infectious Disease Control, National Institute for Public Health and the Environment (RIVM), Bilthoven, The Netherlands; 8 Department of Biomolecular Health Sciences, Faculty of Veterinary Medicine, Utrecht University, Utrecht, The Netherlands; 9 Division of Pharmacology, Utrecht Institute for Pharmaceutical Sciences, Utrecht University, Utrecht, The Netherlands; 10 Danone Nutricia Research, Utrecht, The Netherlands; 11 Department of Paediatric Immunology and Infectious Diseases, Wilhelmina Children’s Hospital/University Medical Center Utrecht, Utrecht, The Netherlands; 12 Department of Neonatology, Wilhelmina Children’s Hospital, University Medical Centre Utrecht, Utrecht, The Netherlands; 13 ReSViNET Foundation, Zeist, The Netherlands

**Keywords:** Breastfeeding, Human milk, Respiratory tract infections, Allergies, Immune development, Antibodies, Human milk oligosaccharides, T cells, Extracellular vesicles, Biobank

## Abstract

**Background:**

Many studies support the protective effect of breastfeeding on respiratory tract infections. Although infant formulas have been developed to provide adequate nutritional solutions, many components in human milk contributing to the protection of newborns and aiding immune development still need to be identified. In this paper we present the methodology of the “Protecting against Respiratory tract lnfections through human Milk Analysis” (PRIMA) cohort, which is an observational, prospective and multi-centre birth cohort aiming to identify novel functions of components in human milk that are protective against respiratory tract infections and allergic diseases early in life.

**Methods:**

For the PRIMA human milk cohort we aim to recruit 1000 mother–child pairs in the first month postpartum. At one week, one, three, and six months after birth, fresh human milk samples will be collected and processed. In order to identify protective components, the level of pathogen specific antibodies, T cell composition, Human milk oligosaccharides, as well as extracellular vesicles (EVs) will be analysed, in the milk samples in relation to clinical data which are collected using two-weekly parental questionnaires. The primary outcome of this study is the number of parent-reported medically attended respiratory infections. Secondary outcomes that will be measured are physician diagnosed (respiratory) infections and allergies during the first year of life.

**Discussion:**

The PRIMA human milk cohort will be a large prospective healthy birth cohort in which we will use an integrated, multidisciplinary approach to identify the longitudinal effect human milk components that play a role in preventing (respiratory) infections and allergies during the first year of life. Ultimately, we believe that this study will provide novel insights into immunomodulatory components in human milk. This may allow for optimizing formula feeding for all non-breastfed infants.

**Supplementary Information:**

The online version contains supplementary material available at 10.1186/s12879-022-07107-w.

## Background

### The burden of respiratory infections

Respiratory infections are one of the major causes of illness in infants, specifically during the first 5 years of life [1]. It was estimated for 2015 that 703,900 children under the age of five died worldwide because of lower respiratory infections [2]. Most respiratory tract infections in children are caused by viral infections, with respiratory syncytial virus (RSV), influenza virus (IFV), parainfluenza (PIV) and metapneumovirus (MPV) being the most commonly found viruses [3, 4]. Each year, about 41.3–112.0 per 1000 children under one year of age were admitted to the hospital for RSV in Europe [5]. This leads to about 28,000 infants requiring medical care for RSV bronchiolitis in the Netherlands [6, 7] of which approximately 2000 require hospitalization. Hospitalisation for respiratory tract infections not only leads to considerable costs, but is also associated with recurrent wheeze, asthma and long term impaired lung function [8–11].

### Breastfeeding protects against infections

Breastfeeding clearly has several benefits for infants, such as the capacity to protect against the risk and impact of neonatal infections [12, 13]. For instance, infants that do not receive exclusive breastfeeding are at a higher risk of hospitalization in early life in relation to a wide range of common infections [14]. Exclusive breastfeeding until the age of four months followed by partial breastfeeding is associated with both reduction in gastrointestinal and respiratory infections [15, 16]. Infants admitted to the hospital with RSV infection are less likely to have been breastfed [10, 17]. In addition, in a recent meta-analysis, one out of the eight risk factors for RSV-induced acute lower respiratory infection in children was “no breastfeeding”, with an odds ratio of 2.24 (95% CI 1.56–3.20) [18]. A similar protective effect of breastfeeding was observed for other common respiratory pathogens like influenza and invasive pneumococcal disease [19, 20].

A meta-analysis strongly favoured breastfeeding over formula feeding to reduce the risk of gastrointestinal infections (i.e. 0.36 (95% confidence interval (CI) 0.3 to 0.41)) [12]. This finding was supported by another meta-analysis that described a higher infant mortality due to diarrheal disease in children who were not exclusively breastfed the first six months of life [21].

The protective effect of breastfeeding on the development of allergic disease is less well pronounced compared to infectious diseases. In a meta-analysis it was found that breastfeeding protects from asthma at ages five to eighteen, eczema until the age of two and for allergic rhinitis until the age of five [22]. Similar results were seen in other studies, for instance the CHILD study that found that direct breastfeeding offers the best protection from asthma during the first three years of age [23, 24]. However, there are also other studies demonstrating that there is even in increased risk on (mild) allergic diseases when children are being breastfed [25–27]. It is particularly hard to evaluate the effect of breastfeeding on the development of allergic diseases, as different definitions are used for allergic disease and different comparisons in duration of lactation [28]. Since the composition of breastfeeding is variable, it may well be that only some components in breastfeeding are protective against allergic diseases and not breastfeeding itself [28]. This hypothesis is supported by the findings of Lodge et al. where groups of human milk oligosaccharide (HMOs) were associated with an increased or reduced change of allergic diseases development in infants [29]. This suggests that breastfeeding contains protective components that may have immunomodulatory capacity.

### Protective components in breastfeeding

Several components within human milk have been linked to the risk of infection during the first year of infancy. Primarily, antibodies in human milk are acknowledged to play a pivotal role protecting infants against various pathogens. Yet, it is still unclear which antibody repertoire would be favourable and what the effect of antibodies is compared to other human milk components. Breakey et al. demonstrated that high IgA antibody titres are linked to a reduction in infectious episodes whereas Mazur et al. described that RSV-specific maternal in breastfeeding IgG but not IgA antibodies are linked to reduction in the risk of RSV infections [30, 31]. Next to pathogen-specific antibodies human milk contains many more components with a protective capacity. For instance, Ramani et al. showed that higher concentrations of the HMO 2’-fucosyllactose (2’-FL) was associated with symptomatic rotaviral infections whereas in another study the presence of 2’-FL was associated with relative protection from respiratory infections [32, 33]. Addition of specific prebiotic oligosaccharide mixtures to infant formulas was shown to decrease the proportion of infections and allergic diseases in children when compared to the standard infant formula [34–38]. In vitro data suggests that HMOs exert their effects through various mechanisms. For instance, HMOs are known to influence interactions between immune cells (e.g. dendritic and T cells), stimulate mucosal barrier and immune system maturation, modulate immune responses following the formation of antigen–antibody complexes, and may affect the immune response following the formation of (endogenous) antibody-antigen complexes [39–44]. HMOs may also influence the gut microbiome of the infant. Breatsfed children usually have a gut microbiome dominated by *Lactobacillus* species and bifidobacteria compared to infant formula fed children [45, 46] Moreover, Ramani et al. fund that LNT (Lacto-N-tetraose) and 6’SL (6′-Sialyllactose) were both associated with *Enterobacter* and *Klebsiella* species [32]. In addition to pathogen-specific antibodies and HMOs, human milk contains many more components, including immune cells and extracellular vesicles (EVs) [47–51]. Several miRNA detected in human milk EVs were immune-related miRNAs [52]. Activated and memory T cells seem to be enriched in early human milk samples and samples from infected mothers, T cells also seem to increase expression of immune proteins during maternal infection [53].

### The PRIMA human milk cohort

Despite decades of development and improvement of infant formulas, formula fed children are still more susceptible to infections early in life [12]. It is largely unknown what impact the different human milk components have on immune development and how they reduce the susceptibility to infections and allergic diseases. Therefore, we initiated the PRIMA human milk cohort in which we will examine the components in human milk that may protect children from infections, in particular respiratory infections, and allergic diseases.

The added value of this cohort is its strong focus on infectious diseases and allergic disease combined with a detailed immunological analyses of the samples on various components (Additional file 2: supplemental protocol). Added to that, we will collect prospective data with longitudinal breastfeeding sampling in n = 1000 children, spread out over multiple winter seasons.

## Methods

### Objectives

#### Primary objective

To identify components in human milk associated with decreased risk of parent-reported medically attended respiratory tract infections (MARI) during the first year of life in healthy term breastfed infants.

#### Secondary objectives

- To identify which components, or combination of components, in human milk are protective against any parent-reported (medically attended) and physician-reported infections during the first year of life.
- To identify which components, or combination of components, in human milk are protective against the development of parent-reported, physician-reported allergic diseases during the first year of life.
- To identify (within a subgroup) which routes are involved in protection transferred from mother to child (human milk, cord blood and amniotic fluid) and are protective against any parent-reported, physician-reported infections/allergic diseases during the first year of life.
- To examine the underlying mechanism of action of the components that have been identified to be protective against MARI, other infections and/or allergic diseases.

### Outcomes

#### Primary clinical endpoint

The number of parent-reported medically attended respiratory infections during the first year of life.

#### Secondary clinical endpoints

- The number of physician-reported medically attended respiratory infections during the first year of life.
- The number of other parent-reported infections during the first year of life.
- The number of parent-reported allergic diseases (e.g. atopic dermatitis and egg/cow’s milk allergies) during the first year of life.
- The number of other physician-reported infections during the first year of life.
- The number of physician-reported allergies during the first year of life.
- The number of antibiotic courses during the first year of life

### General study design

**Fig. 1:**
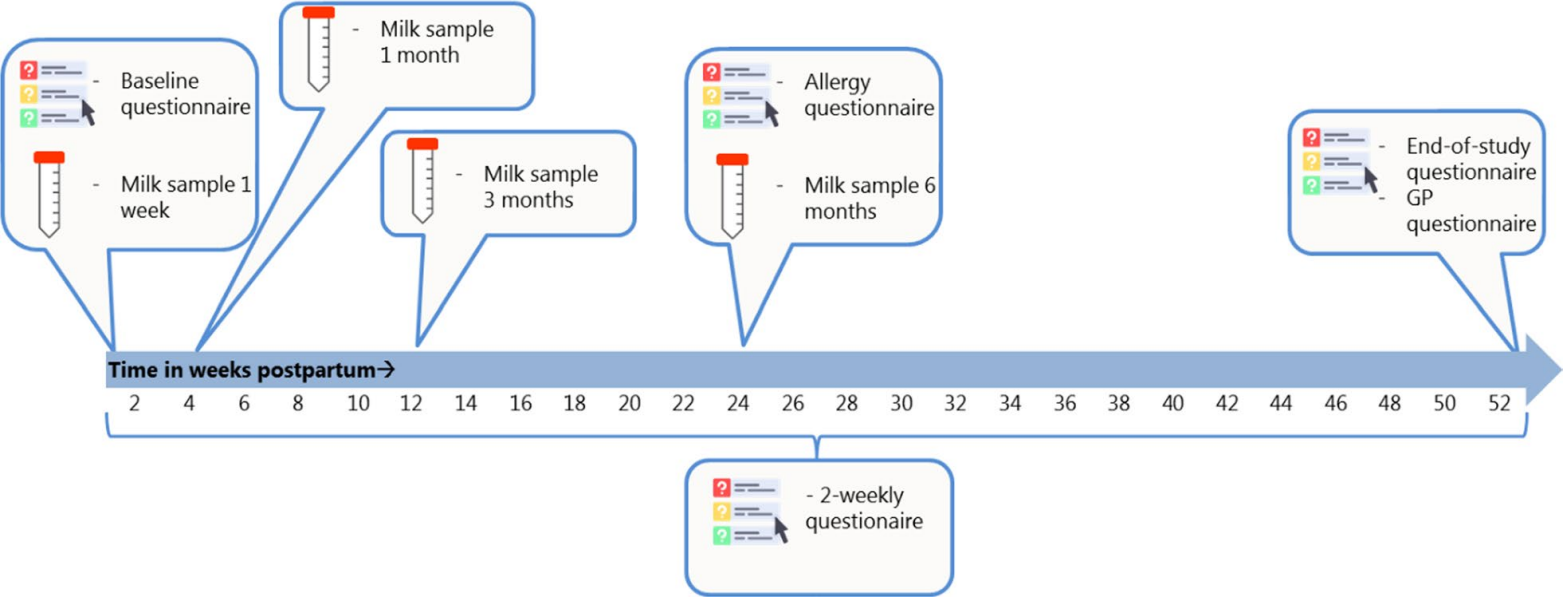
Overview of sampling and timing of questionnaire: overview of timing sample collection and questionnaires sent to parents

In this prospective, observational cohort we will recruit a total of 1000 mother–child pairs during the first month postpartum. The study population will consist of healthy mothers and children in the Utrecht region, the Netherlands, that already started breastfeeding. Human milk samples will be collected at one week, one month, three months and six months if possible (Fig. 1). These timepoints span colostrum/transitional milk sample (within first week postpartum), early mature (one month sample) and late mature samples (three and six months) [54, 55]. During the first year of follow up, mothers will report their and their infant’s susceptibility to infectious and allergic diseases by completing digital questionnaires sent by email every two weeks until the child reaches one year of age. After the infant has reached the age of one year, their general practitioner will be contacted to collect clinical data on infectious episodes, e.g. antibiotic use and sputum cultures during the first year of life (Additional file 1: Fig S1). In a nested subgroup (n = 20), we will collect additional samples of maternal blood, cord blood, amniotic fluid and neonatal saliva and neonatal faecal samples. This subgroup will be used as a pilot to study the effect of all different routes through which mothers may transfer immune active components.

The milk sample composition will be linked to clinical data in order to identify the components or combination of components associated with medically attended respiratory infections, other infections and/or allergies. The human milk samples will be stored in the biobank facility of the UMC Utrecht for future research, securing quality of sample storage. The study recruitment started in March 2019 and is still recruiting participants. The expected end date of recruitment will be in June 2023. Consequently the study will end one year later in June 2024. The study is registered in The Netherlands Trial Register (https://www.trialregister.nl/) under protocol number NL9056.

### Study participants

Participants are recruited at the obstetric wards of the University Medical Centre Utrecht (tertiary centre) and the Diakonessenhuis hospital Utrecht (secondary centre). Participants are recruited in two different ways. Mothers that are admitted to the obstetric wards of either hospitals, will be informed by their midwives about the study. Mothers are asked approval to share their contact details with the researchers of the PRIMA cohort. Mothers that gave consent to share their contact details are contacted consequently.

Mothers that give birth in the primary care birth centre of the University Medical Centre Utrecht will receive a letter from their midwives at discharge. This letter contains information about the study and the possibility to opt out to be contacted by the researchers. 48 h after discharge, researchers will receive only the contact details of those parents that did not indicate objection to be contacted.

Mothers are screened by eligibility by the researchers during the first phone call. Additional information about the study will be provided and whenever parents are willing to participate, they will be send the patient information folder and the first house visit to collect milk and informed consents are scheduled.

Inclusion is performed within the first week postpartum. An overview of inclusion criteria and study activities overview can be found in Table 1 and Additional file 1: Fig. S1, respectively.

**Table 1:** Eligible criteria for the PRIMA human milk cohort

	Inclusion criteria	Exclusion criteria
Parents	All mother–child pairs that live in a proximity to Utrecht	Acquired or innate immune deficiencies
	Parents currently breastfeeding and intending to continue breastfeeding until at least 3 months postpartum	Insufficient control of the Dutch language
Infant		Severe cardiac or pulmonary disorders or other severe organ diseases
		Severe prematurity defined as GA < 32 weeks

### Data collection

At inclusion, parents will fill out a baseline questionnaire to collect data on (Fig. 1):

- Perinatal and pregnancy health characteristics.
- Family demographics.
- Health status parents.
- Vaccination status of the mother.
  After inclusion, participants will receive a questionnaire every 2 weeks collecting data about:
- Infectious episodes, allergic symptoms, feeding habits, day-care attendance and growth characteristics of infant.
- Doctor visits, diagnoses made and medication prescribed to infant.
- Medication use and health status mother
- Any changes in demographics.

At 6 and 12 months, an additional questionnaire will be added to the 2-weekly questionnaire to record any allergic disease manifestations (Fig. 1). This questionnaire is based on the EuroPrevall infant cohort questionnaires that were designed to investigate the prevalence of allergies in the European population [56]. At twelve months, the vaccination status of the infant, changes in household (e.g. number of children or pets) and more detailed information about any hospital admission of the infant are recorded (Fig. 1).

### Sample processing

Samples are collected by participants at home, either by hand or by breast pump. Within 24 h, study personnel will collect the samples and will note time of expression, method of expression and whether milk was collected before, during or after a feeding or whether it was a whole feeding sample collected. Participants will store the milk samples in their own fridge until study personnel collects and transfers the samples on ice to the UMC Utrecht laboratory. After milk expression, milk is stored on ice or in the fridge until processing. We record what time the sample was expressed and whether it is a whole milk sample or milk collected only before or after a whole feeding. Within 24-h after collection, samples will be processed according to the protocol of Zonneveld et al., using 600 times gravity instead of 3000 times gravity to preserve living cells (Additional file 2: Fig. S2) [57]. After processing, milk supernatant and cream fractions will be stored in cryotubes at – 80 °C. Fresh cell fractions will be used for T cell assays. Antibody and HMO composition is measured in milk supernatant. Samples that will be used for EV analysis, will be processed within 30 min after collection following the above mentioned protocol of Zonneveld et al. [57]. To ensure samples are processed within 30 min, only participants that live in close proximity to the lab and that had sufficient milk production form the first week postpartum are selected for EV isolation. Study personnel is present at moment of milk collection and transfer samples within 20 min at 37C6 to our laboratory. Total time of transport is recorded for each sample.

### Sample analysis

The milk samples collected in this cohort will be analysed for antibody and oligosaccharide composition (fraction D, Additional file 2: Fig. S2). EV and T cell composition will be analysed in subgroups. The HMO composition is determined by mass spectrometry (MS) based approaches. Recent advances in Liquid chromatography mass spectrometry (LC–MS) enables detection of a vast range of HMOs and to determine relative HMO concentration [58]. A recently published LC–MS assay has shown to successfully detect semi-quantitively human milk tri-, tetra-, penta- and hexaoses in a single LC–MS assay run[58].

Next to HMOs, samples will be analysed for antibody composition using a bead-based multiplex assay. It has been previously shown that bead-based multiplex assays can reliably measure antibody titres against several pathogens in blood and are an effective means of assessing immunity in a population [59–63]. After optimization, we will use a bead-based multiplex to determine pathogen specific antibodies, e.g. Severe acute respiratory syndrome coronavirus 2 (SARS-CoV-2), Cytomegalovirus (CMV), Bordetella pertussis, pneumococcus, RSV and influenza-specific antibodies [59–62, 64]. Additionally, we will also use a bead-based multiplex assay to determine the concentration of all human antibody isotypes and their subclasses [59–62]. Preliminary data showed that it is also feasible to use these assays in human milk samples, though we will have to optimise these multiplex assays further for the measurement of human milk antibody titres.

In a randomly selected subgroup, we will analyse T cell profiles and functional skewing with flow cytometry as well as their transcriptome and T-cell receptor (TCR) repertoire using single-cell RNA/TCR- sequencing.

### Statistical considerations

We base our sample size calculation on the variation in 2’-FL concentration, as this is one of the most abundant HMOs present in human milk [65]. The sample size of 1000 participants has an 80% power to detect a ratio of the means of 0.91 in those with vs. those without a parent-reported medically attended respiratory infection using a two-sided two-sample t-test, assuming that 10% of the cohort participants have a parent-reported medically attended respiratory infection (N = 100) and that the 2’-FL HMO concentration in the population has a mean of 3 g/L, a standard deviation of 1 g/L, and a normal distribution [65]. The significance level (alpha) is 0.05. The sample size was calculated using Power Analysis for Sample Size (PASS) 2008.

The primary aim of this study is to identify components in human milk associated with decreased risk of parent-reported MARI. To this end we will analyse the association between 2’FL concentration and the MARI during the first year of life using regression analysis. The possible association between 2’-FL concentrations and the number of parent-reported medically attended respiratory infections during the first year of life will be determined using multivariable Poisson regression analysis, adjusting for potential confounders. Potential confounders will be identified by a literature search, complemented by expert knowledge. We will analyze whether the moment of maternal atopy, birth month, duration of lactation postpartum, season, socio-economic status and ethnicity modify the effect of HMOs on the number of respiratory infections.

For our secondary analysis, we will analyse the relationship between HMOs (other than 2’FL) or antibodies and MARI in a similar fashion as 2’FL using regression analysis and Poisson analysis.

For the secondary analysis, the statistical analysis plan will be based on the cohort of 1000 mother child pairs, providing milk samples as well as consecutive questionnaire data related to parent-reported medically attended respiratory infection (MARI). Based on the parent reported respiratory tract infection symptoms, the number infectious episodes during the first year of life will be defined and separates the cohort into two groups (high frequency of infection vs low frequency of infection). We will analyze the relationship between HMOs and antibodies and MARI with a machine-learning based multivariate analysis. We will use recursive ensemble feature selection (REFS) algorithm [66] to reduce the variables to the minimum necessary set to differentiate between the two groups (signature). REFS has been applied successfully to different types of complex biological data and has been shown to have better accuracy [67] than univariate methods and stable enough between datasets [68]. Finally, to validate our results we will use metrics such as area under the curve (AUC) and receiver operating characteristic (ROC) curve [69, 70] to validate the diagnostic prediction using the found signature. A similar approach can be followed regarding studying the relation between parent-reported, physician-reported allergic diseases during the first year of life (high frequency of allergy vs low frequency of allergy) and HMOs and antibodies in human milk.

In case there is loss to follow-up because parents stop to report symptoms, we will use the data retrieved from the general practioner to estimate incidence of MARI. If this is not possible, we will impute the missing data using multiple imputation. We will impute missing data using multiple imputation.

## Discussion

Respiratory infections are still a major cause of burden of disease and child death, RSV being the most common found pathogen [4, 17, 71]. In 2015, the worldwide death of 118,200 children under five years of age were attributed to RSV [72]. Protection from respiratory infections will not only lead to a reduction in childhood mortality or other complications, but protection from viral infections is also associated with reduction of other diseases, like recurrent wheeze during first year of life and even asthma [73, 74]. Infants can be protected by vaccinations either given early after birth or by maternal vaccination during pregnancy [75]. These vaccinations elicit a pathogen-specific immune response, offering protection to these specific pathogen only [75]. Currently, no effective (maternal) vaccination is available in the clinic to protect from RSV infections. An important and validated strategy to reduce the number of respiratory infections is breastfeeding [12, 17]. Despite all efforts taken, still, the number of children being exclusively breastfed for six months remains low in some regions. In 2006–2012, only an estimated 25% of infants in the World Health Organisation (WHO) European Region were exclusively breastfed for the first 6 months, which is far below the WHO recommendation of 6 months exclusive followed partial breastfeeding until the age of two.

Understanding the composition of breast milk in relation to disease susceptibility in early life will guide the development of much needed interventions for infants that cannot be exclusively breast fed. At the present moment, there is a lack in knowledge about the specific components or combination of components present in human milk that support the protective capacity against infections. In order to be able to improve safety and efficacy of infant formulas, it first has to be established which components including their underlying mechanism of action in breastfeeding are essential to protect against infections.

The PRIMA human milk cohort is designed to study human milk components in a novel public–private collaboration. The PRIMA human milk cohort will be one of the largest cohorts combining both longitudinal human milk samples with prospective clinical data collection. This initiative will offer a unique possibility to add scientific understanding regarding the protective effect and influence of specific human milk derived components on the protection against respiratory infections and allergies early in life. Data and samples from this cohort will be used to study the underlying mechanism of several components in human milk which might lead to the development of new concepts/hypotheses for the management and therapy of immune related disorders such as allergies and infections.

## Supplementary Information

**Additional file 1: Figure S1.** Study activities overview: overview of all study activities. **Figure S2.** Sample processing in PRIMA human milk cohort: A) Human milk samples are centrifuged for 10 min at 600 g in order to remove the cream (layer on top, indicated in yellow) and cells (collected in pellet on the bottom, indicated in dark grey). B) Milk supernatant is collected by pipetting through cream layer without disturbing the cell pellet and subsequently transferred into a new tube. C) Collected 600 g supernatant is again centrifuged for another 10 min at 600 g. D) Milk supernatant is collected by pipetting through the residual cream and transferred to and aliquoted into clean cryotubes, and stored at -80 °C until further analysis. E) The cream layer is removed without disturbing the cellular pellet and transferred into cryotubes stored at -80 °C for further analysis. F) 20 mL PBS is added to rinse and cells are spun at 600 g for 10 min, after which the supernatant is discarded and cells are kept on ice until further processing for analysis G). Milk EVs are processed further according to the Zonneveld protocol at 3000 g.

**Additional file 2.** PRIMA research protocol METC Utrecht version 4A: this file contains the protocol including the novel additions for collection of dietary information from mother.

## Data Availability

As the current manuscript describes the study protocol and no other data, we do not have any raw data to share at the moment.

## Abbreviations

EV: Extracellular vesicle
RSV: Respiratory syncytial virus
IFV: Influenza virus
PIV: Parainfluenza
MPV: Human metapneumovirus
CI: Confidence interval
HMO(S): Human milk oligosaccharide(s)
IgA: Immunoglobulin A
IgG: Immunoglobulin G
2’FL: 2’-Fucosyllactose
MARI: Medically attended respiratory infection
MS: Mass spectrometry
LC–MS: Liquid chromatography mass spectrometry
SARS-CoV-2: Severe acute respiratory syndrome coronavirus 2
CMV: Cytomegalovirus
TCR: T-cell receptor
PASS: Power analysis for sample size
WHO: World Health Organisation
GA: Gestational age
