# Supplementary material for: Analysing the protection from respiratory tract infections and allergic diseases early in life by human milk components: the PRIMA birth cohort
Source: BMC Infect Dis. 2022 Feb 14;22:152. doi: 10.1186/s12879-022-07107-w (PMC8842741; doi:10.1186/s12879-022-07107-w)
Supplement: Supplementary file 1 — Additional file 1: Figure S1. Study activities overview: overview of all study activities. Figure S2. Sample processing in PRIMA human milk cohort: A) Human milk samples are centrifuged for 10 min at 600 g in order to remove the cream (layer on top, indicated in yellow) and cells (collected in pellet on the bottom, indicated in dark grey). B) Milk supernatant is collected by pipetting through cream layer without disturbing the cell pellet and subsequently transferred into a new tube. C) Collected 600 g supernatant is again centrifuged for another 10 min at 600 g. D) Milk supernatant is collected by pipetting through the residual cream and transferred to and aliquoted into clean cryotubes, and stored at -80 °C until further analysis. E) The cream layer is removed without disturbing the cellular pellet and transferred into cryotubes stored at -80 °C for further analysis. F) 20 mL PBS is added to rinse and cells are spun at 600 g for 10 min, after which the supernatant is discarded and cells are kept on ice until further processing for analysis G). Milk EVs are processed further according to the Zonneveld protocol at 3000 g. [file 12879_2022_7107_MOESM1_ESM.docx]

**Figure S1:**

**
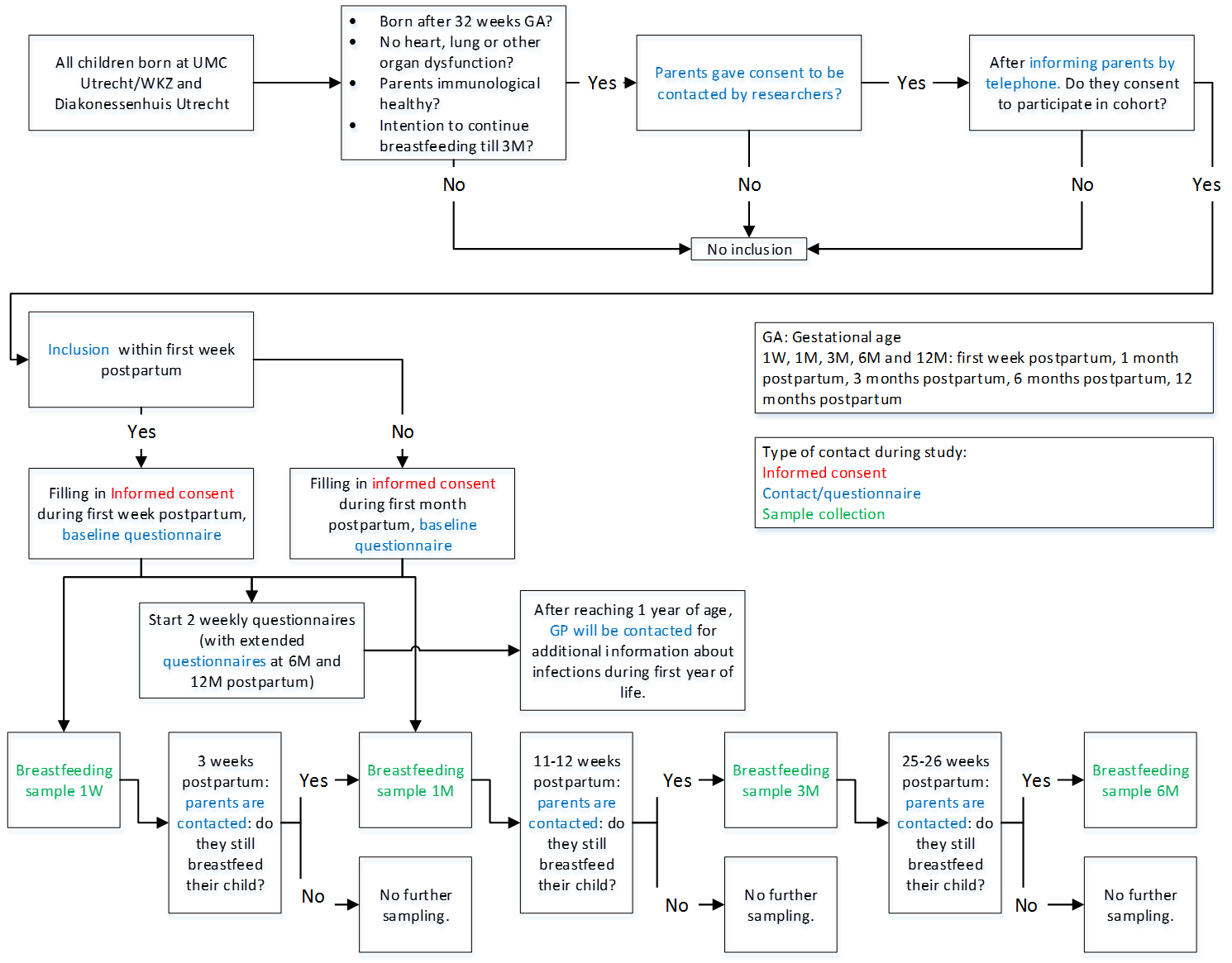
**

**Figure S1. Study activities overview:** overview of all study activities

**Figure S2:**


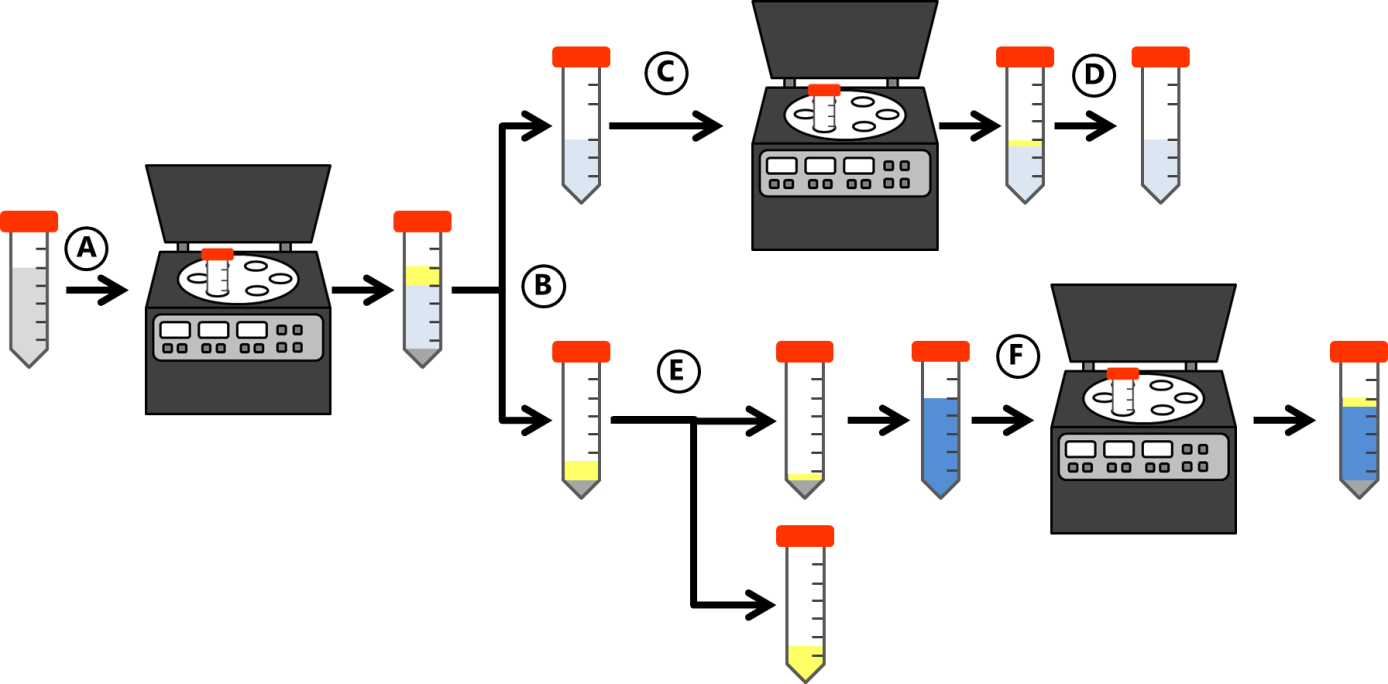


**Figure S2. Sample processing in PRIMA human milk cohort:** A) Human milk samples are centrifuged for 10 minutes at 600 g in order to remove the cream (layer on top, indicated in yellow) and cells (collected in pellet on the bottom, indicated in dark grey). B) Milk supernatant is collected by pipetting through cream layer without disturbing the cell pellet and subsequently transferred into a new tube. C) Collected 600 g supernatant is again centrifuged for another 10 minutes at 600 g. D) Milk supernatant is collected by pipetting through the residual cream and transferred to and aliquoted into clean cryotubes, and stored at -80^o^C until further analysis. E) The cream layer is removed without disturbing the cellular pellet and transferred into cryotubes stored at -80 ^o^C for further analysis. F) 20mL PBS is added to rinse and cells are spun at 600 g for 10 minutes, after which the supernatant is discarded and cells are kept on ice until further processing for analysis G). Milk EVs are processed further according to the Zonneveld protocol at 3000 g.
