# Supplementary material for: Analysing the protection from respiratory tract infections and allergic diseases early in life by human milk components: the PRIMA birth cohort
Source: BMC Infect Dis. 2022 Feb 14;22:152. doi: 10.1186/s12879-022-07107-w (PMC8842741; doi:10.1186/s12879-022-07107-w)
Supplement: Supplementary file 2 — Additional file 2. PRIMA research protocol METC Utrecht version 4A: this file contains the protocol including the novel additions for collection of dietary information from mother. [file 12879_2022_7107_MOESM2_ESM.docx]

**PRIMA RESEARCH PROTOCOL**

**Protecting against Respiratory tract Infections through human Milk Analysis**

**
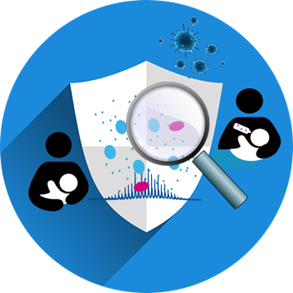
**

**PROTOCOL TITLE ‘**Protecting against Respiratory Infections through human Milk Analysis’

| **Protocol ID** | **PRIMA** |
| --- | --- |
| **Short title** | **Protecting against Respiratory Infections through human Milk Analysis** |
| **EudraCT number** | ***Not applicable*** |
| **Version 4A** |  |
| **Date 8-7-2021** |  |
| **Coordinating investigator/project leader** | ***A.H. Hellinga, PhD-student*** |
| **Principal investigator(s) (in Dutch: hoofdonderzoeker/ uitvoerder)** | ***Prof. L.J. Bont, MD Ph, PI RSV research group***  ***J.H.W. Leusen, PHD, PI immunotherapy research group***  ***B. Van’t Land, PHD, PI immune development group*** |
|  |  |
| **Sponsor (in Dutch: verrichter/opdrachtgever)** | ***UMC Utrecht*** |
|  |  |
| **Subsidising party** | ***Nutricia Research bv.***  ***Regio Deal Foodvalley*** |
| **Independent expert (s)** | ***Bert Arets, MD PhD*** |
|  |  |
|  |  |
| **Laboratory sites** | ***Laboratory of translational immunology (LTI), dLAB, UMC Utrecht***  ***Nutricia Research bv***  ***Division of Pharmacology l Department of Pharmaceutical Sciences l Utrecht Institute for Pharmaceutical Sciences*** |
|  |  |
| **Pharmacy** | ***Not applicable*** |
|  |  |

**PROTOCOL SIGNATURE SHEET**

| **Name** | **Signature** | **Date** |
| --- | --- | --- |
| **Sponsor or legal representative:**  ***Management of dLAB:***  ***Prof. Dr. R.A.W. van Lier.***  **Head of Department:**  ***Prof. Dr. L. Meyaard*** |  |  |
| **Principal Investigator:**  ***Prof. dr. L.J. Bont*** |  |  |

**TABLE OF CONTENTS**

1. INTRODUCTION AND RATIONALE 12

2. OBJECTIVES 16

3. STUDY DESIGN 17

4. STUDY POPULATION 17

4.1 Population (base) 17

4.2 Inclusion criteria 18

4.3 Exclusion criteria 18

4.4 Sample size calculation 19

Study A: 19

5. METHODS 19

5.1 Study parameters/endpoints 19

5.1.1 Main study parameter/endpoint 19

Parent-reported medically attended respiratory tract infection during the first year of life 19

5.1.2 Secondary study parameters/endpoints (if applicable) 19

5.1.3 Other study parameters (if applicable) 20

5.2 Study procedures 20

5.3 Withdrawal of individual subjects 22

5.3.1 Specific criteria for withdrawal (if applicable) 22

5.4 Replacement of individual subjects after withdrawal 22

5.5 Follow-up of subjects withdrawn from treatment 22

5.6 Premature termination of the study 22

6. SAFETY REPORTING 22

7. STATISTICAL ANALYSIS 22

7.1 Primary study parameter(s) 22

postpartum that sample is collected). 23

7.2 Secondary study parameter(s) 23

7.3 Other study parameters 23

7.4 Missing data 23

8. ETHICAL CONSIDERATIONS 23

8.1 Regulation statement 23

8.2 Recruitment and consent 23

8.3 Objection by minors or incapacitated subjects (if applicable) 24

8.4 Benefits and risks assessment, group relatedness 24

8.5 Compensation for injury 25

8.6 Incentives (if applicable) 25

9. ADMINISTRATIVE ASPECTS, MONITORING AND PUBLICATION 25

9.1 Handling and storage of data and documents 25

9.2 Monitoring and Quality Assurance 26

9.3 Amendments 26

9.4 Annual progress report 26

9.5 Temporary halt and (prematurely) end of study report 27

9.6 Public disclosure and publication policy 27

10. REFERENCES 27

**LIST OF ABBREVIATIONS AND RELEVANT DEFINITIONS**

| **ABR** | **General Assessment and Registration form (ABR form), the application form that is required for submission to the accredited Ethics Committee; in Dutch: Algemeen Beoordelings- en Registratieformulier (ABR-formulier)** |
| --- | --- |
| **AE** | **Adverse Event** |
| **AR** | **Adverse Reaction** |
| **CA** | **Competent Authority** |
| **CCMO** | **Central Committee on Research Involving Human Subjects; in Dutch: Centrale Commissie Mensgebonden Onderzoek** |
| **CV** | **Curriculum Vitae** |
| **DSMB** | **Data Safety Monitoring Board** |
| **EU** | **European Union** |
| **EudraCT** | **European drug regulatory affairs Clinical Trials** |
| **GCP** | **Good Clinical Practice** |
| **GDPR** | **General Data Protection Regulation; in Dutch: Algemene Verordening Gegevensbescherming (AVG)** |
| **IB** | **Investigator’s Brochure** |
| **IC** | **Informed Consent** |
| **IMP** | **Investigational Medicinal Product** |
| **IMPD** | **Investigational Medicinal Product Dossier** |
| **METC** | **Medical research ethics committee (MREC); in Dutch: medisch-ethische toetsingscommissie (METC)** |
| **(S)AE** | **(Serious) Adverse Event** |
| **SPC** | **Summary of Product Characteristics; in Dutch: officiële productinformatie IB1-tekst** |
| **Sponsor** | **The sponsor is the party that commissions the organisation or performance of the research, for example a pharmaceutical**  **company, academic hospital, scientific organisation or investigator. A party that provides funding for a study but does not commission it is not regarded as the sponsor, but referred to as a subsidising party.** |
| **SUSAR** | **Suspected Unexpected Serious Adverse Reaction** |
| **UAVG** | **Dutch Act on Implementation of the General Data Protection Regulation; in Dutch: Uitvoeringswet AVG** |
| **WMO** | **Medical Research Involving Human Subjects Act; in Dutch: Wet Medisch-wetenschappelijk Onderzoek met Mensen** |

**SUMMARY**

**Rationale:**

Study A:

Infants who are not breastfed have a higher risk of hospitalization in early life in relation to a wide range of common infections (Ajetunmobi2015). Exclusive breastfeeding until the age of 4 months followed by partial breastfeeding is associated with reduction in respiratory and gastrointestinal infections (Walker2015, Duijts2010). For example, infants admitted to hospital with respiratory syncytial virus (RSV) infection are less likely to have been breastfed (Ajetunmobi2015, Blanken 2018). This suggests that breast milk contains protective components against respiratory tract infections, which lack in infant formula. Although infant formula has been developed over many decades into adequate nutrition for those infants who cannot receive human milk, even modern infant formulas lack many components made by each mother for the immune imprinting of her baby, such as specific antibodies (based on the immunologic history of the mother) and human milk oligosaccharides (HMOS). The provision of secretory mixtures of HMOS, have been associated with direct protection against infections (Laucirica2017). The protective effects of breast milk are, in part, due to antibodies and in part due to other factors, including oligosaccharides. Identification and confirmation of the compounds and their mechanisms by which they offer protection against infections is critical to be able to design new and improved formula products. In addition, compounds, or combination of compounds, that seem to have a protective effect, could be used as novel strategies to treat pneumonia and other respiratory tract infections.

Study B:

There is a growing interest in the use of maternal vaccinations during pregnancy and several maternal vaccinations are at this moment studied, e.g. against Bordetella pertussis, influenza, group B streptococcus and RSV (Marchant2017, Abu Raya2017, Heath2017) ). Already, a maternal vaccination against Bordetella pertussis is approved for vaccination during pregnancy and is currently implemented in the UK (Amirthalingam2016). From December 2019 onwards, all pregnant women in the Netherlands will be offered a Bordetella pertussis vaccination at 28 till 32 weeks gestational age (Rijksoverheid). Although it is generally believed that the protective effect of maternal vaccination is derived from placental transfer of antibodies, definitive evidence whether placental transfer or transfer of immunity through breastfeeding or amniotic fluid is absent (Marchant2017, Pou2019). Moreover, maternal vaccination studies in mice demonstrated that transfer of antibodies through breastfeeding and not placental transfer is of major importance to protect offspring (Gaillard2017,Caballero-Flores2019). Further mechanistic insights into maternal immunity transfer will be essential for further development of maternal vaccinations. Therefore we plan to perform a substudy (afterwards called study B) within the PRIMA human milk cohort. In this cohort (n=20) we will collect additional samples next to the breastfeeding samples and the questionnaires. We will sample cord blood, amniotic fluid, maternal blood sample and saliva samples of the participating children. Cord blood samples and the amniotic fluid samples will be compared to the composition of antibodies and HMOS in human milk, as it is known they contain HMOs and/or antibodies as well (Jacobino2016, Hirschmugl2019). The maternal blood samples will be used to observe the maternal response to Bordetella pertussis revaccination. Likewise, the maternal blood sample will serve as a golden standard to see if there is any kind of selection of antibody transfer through one of the three media (i.e. cord blood, human milk or amniotic fluid).

To evaluate the influence of each individual route of maternal immune transfer on immune development, we will also collect saliva samples. We have a particular interest in the development of the antibody repertoire in children enrolled in this substudy. To further evaluate the effect of the maternal Bordetella pertussis vaccination on the immune development in infants, saliva samples will be collected during the house visits (i.e. at 1 week, 1 month, 3, 6 and 12 months postpartum). Saliva samples have demonstrated to be a reliable representation of antibody serum repertoire, and are considered less invasive then blood samples (Litt2006, Hetteger2019).

Study C:

The PRIMA cohort (i.e. study A) is performed to identify which components, or combination of components, in human milk are protective against respiratory tract infection in infants. The composition of these components in human milk is influenced by many factors. Some of these factors cannot be influenced, such as the origin of the mother, whereas other factors can be influenced, such as the diet of a lactating woman (Bravi et al., 2016; Munblit et al., 2014). As an example, an observational study with Finnish lactating woman found that a breast milk sample 3 months postpartum of women who consumed fatty fish in the two days before sample collection had a 34% increase of omega-3 fatty acids, compared to breast milk of women who did not consume fatty fish (Mäkelä et al., 2013). For other components, such as HMO composition, the influence of the maternal diet has been studied very limited but the study of Quin and collegeagues found a positive correlation between fruit intake and unsaturated fatty acids consumed on the day before breast milk sampling with various HMOs in breast milk (Quin et al., 2020). Even more importantly, although there are strong indications that the maternal diet influences the breast milk composition, it is not studied what the consequential effect maternal diet has on infants’ health outcomes. However, it is known that the omega-3 polyunsaturated fatty acid docoheaxenoic acid (DHA) have an increased capacity to phagocytise and have an increased microbicidal activity (Hachey et al., 1989; Nasser et al., 2010). Also for the HMOs in breast milk, there is increasing attention for and evidence of their antimicrobial, antiviral and prebiotic capacity (Ayechu-Muruzabal et al., 2018; Morozov et al., 2018). The impact of this on the intestinal microbiota composition might also impact the development of respiratory tract infections and/or allergies via the gut-lung axis (Hilty et al., 2010; Kanmani et al., 2017; Remot et al., 2017; Schuijt et al., 2016). Together, this shows that there is a strong need to study the impact of the maternal diet on breast milk composition, and how this consequently influences the development of respiratory tract infections and/or allergies in the first year of life. Therefore, the cohort should be expanded with dietary assessment of the mother’s diet, and sample infants’ saliva and feces.

**Objective**:

**Study A:**

Main objective:
To identify which components, or combination of components, in human milk and cord blood that are protective against respiratory tract infection during the first year of life and to unravel their underlying mechanisms.

Secondary objective 1:

To identify which components, or combination of components, in human milk are protective against other infections during the first year of life and to unravel their underlying mechanisms.

Secondary objective 2:

To identify which components, or combination of components, in human milk are protective against the development of allergies during the first year of life and to unravel their underlying mechanisms.

Secondary objective 3:

To obtain insight in the gut discomfort and crying of infants in the first 6 months of life.

**Study B:**

Secondary objective 4:

To obtain insight in the transfer of maternal immunity to human milk, amniotic fluid and cord blood.

**Study C:**

Secondary objective 5:

To identify the long-term and short-term influence of the maternal diet on the breast milk composition.

Secondary objective 6:

Link the long-term and short-term influence of the maternal diet to the clinical outcome of respiratory tract infection and/or allergies during the first year of life.

Secondary objective 7:

To derive hypotheses about potential mechanisms if the maternal diet-influenced components in human milk are protective against respiratory tract infection and/or of allergies during the first year of life.

Secondary objective 8:

To identify the association between long-term and short-term influence of the maternal diet on the microbiota composition of a 3-month old infant, and its consequence for the risk of developing respiratory tract infections and/or allergies in the first year of life.

Secondary objective 9:

To identify the antibody repertoire and viral genome in saliva samples from 3 month-old

Secondary objective 10:

To link the antibody repertoire, viral genome load and possible other immune markers in saliva samples with feces microbiota composition, and how this relates to the risk of developing respiratory tract infections in the first year of life.

**Study A:**

**Study design:** Observational birth cohort study

**Study population:** healthy mother-child dyads recruited during the first month postpartum.

**Intervention (if applicable)**: none.

**Main study parameters/endpoints:** The main study parameter is the number of MARI’s (Medically Attended Respiratory tract Infections) during the first year of life. This will be measured using questionnaires that parents have to fill in every two weeks. In the same questionnaire, the gut discomfort and crying time of the infant is assessed for the first six months.During the 6-month and 12-month questionnaire, questions about allergy development are added to the questionnaire.

In addition to the questionnaire, we will also collect samples both from the mother as from the child.

Human milk samples will be collected from the mothers during the first week postpartum and at 1, 3 and 6 months postpartum. In human milk, we will measure the antibody titters (both isotype as well as pathogen specific), oligosaccharide composition and immune cell repertoire. A subgroup will be used to analyse extra cellular vesicles (n=15). Since the extracellular vesicle analyses requires bigger sample volumes (at least 5mL sample is required for vesicle analyses, preferably more), only mothers that donate a large amount of human milk at the first week postpartum will be analysed for extracellular vesicles.

Amniotic fluid and cord blood and maternal blood samples will be collected in a subgroup of 20 mother-child pairs. Cord blood and amniotic fluid will be collected within the first hours postpartum from the umbilicus. Maternal blood samples will be collected during the first week postpartum. Blood and amniotic fluid samples will be analysed on antibody, oligosaccharide and extracellular vesicle composition and immune cell repertoire

**Study B:**

**Study design:** Observational birth cohort study

**Study population:** healthy mother-child dyads recruited during pregnancy.

**Intervention (if applicable)**: none.

**Main study parameters/endpoints:**

The main study parameter is the number of MARI’s (Medically Attended Respiratory tract Infections) during the first year of life. This will be measured using questionnaires that parents have to fill in every two weeks.

Human milk samples will be collected from the mothers during the first week postpartum and at 1, 3 and 6 months postpartum. In human milk, we will measure the antibody titters (both isotype as well as pathogen specific), oligosaccharide composition and immune cell repertoire. At 1 week, 1 month, 3 months, 6 months and 12 months postpartum saliva sample of the infants will be collected. Saliva samples will be analysed on antibody repertoire.

Additionally, amniotic fluid, cord blood, infant saliva and maternal blood samples will be collected. Cord blood and amniotic fluid will be collected within the first hours postpartum from the umbilicus. Maternal blood samples will be collected during the first week postpartum. Blood and amniotic fluid samples will be analysed on antibody, oligosaccharide and extracellular vesicle composition and immune cell repertoire.

**Study C:**

**Study design:** Observational birth cohort study

**Study population:** healthy mother-child dyads recruited during pregnancy.

**Intervention (if applicable)**: none.

**Main study parameters/endpoints:**

The main study parameters/endpoints of study A will also account for sub-study C.

Additionally, a main study parameter is the diet of lactating women. The dietary pattern (long-term) will be assessed by a food frequency questionnaire (FFQ) at 2 month and 4 month post-partum. Additionally, we will assess the short-term influence of the diet on breast milk composition by a 24-h recall on the day before the collection of a 1-month and 3-month human milk sample.

In addition to the questionnaires, we will also collect samples both from the mother (human milk) as from the child (saliva and feces):

In the collected human milk samples (1 week, 1 month, 3 and 6 months postpartum) we will perform additional analyses, such as to measure fatty acid composition, protein and amino acid composition, fat- and water-soluble vitamins and other fibres (than HMOs).

The feces will be collected from infants at 3 months postpartum. The feces will be analysed for microbial composition.

The saliva will be collected from infants at 3 months postpartum. The saliva will be analysed for the antibody repertoire and viral genome.

**Nature and extent of the burden and risks associated with participation, benefit and group relatedness:**

**Study A , B and C:**

Breast milk is collected by pump or by manual expression. In case women who do not intend to use a pump, will use a pump, this may result in some discomfort. Burden and risks of expressing milk: expressing milk may cause some discomfort. No specific risks are reported with expressing breastmilk.

Number, timing and amount of samples: We will collect four samples: during the first week post-partum, 1 month, 3 months and 6 months postpartum. We will to collect 5-10mL for each sample.

Questionnaires to assess the MARIs have to be filled in during the trial: 27 (baseline and afterwards 26 questionnaires every 2 weeks (including the longer questionnaires at 6 and 12 months post-partum)). Estimated total time spend on questionnaire is 2-3 hours per participating mother-child dyad spread over 1 year.

**Study B:**

Burden and risk of cord blood samples: Since blood will be collected form the cord blood, we expect no burden or risks arising from cord blood sampling.

Burden and risk of amniotic fluid samples: Since amniotic fluid will be collected in a non-invasive way, we expect no burden or risks arising from amniotic fluidsampling.

Maternal blood sampling: Maternal blood will be collected through a venipuncture. Although considered safe, rarely phlebitis, extravasation of blood, bruising and hematoma forming following venipuncture have been reported. Likewise a venipuncture might cause discomfort (i.e. pain during venepuncture or from the arising hematoma or bruises).

Burden and risk of saliva samples: Since saliva will be collected in a non-invasive way, we expect no burden or risks arising from saliva sampling. A similar methods of saliva collection is used in the SInFoNIA study (NL65543.041.18).

Burden and risk of amniotic fluid samples: No risk or burden to be expected.

**Study C:**

Dietary assessment

The FFQs have to be filled in around 2 months and 4 months postpartum. The 24h-recall is filled in at 1 month and 3 month postpartum. Estimated total time spend on dietary assessment questionnaire is 3 hours per participating mother-child dyad spread over 3 months.

Feces sampling

Feces will be samples by the parent from the diaper of the infant. Since feces will be collected in a non-invasive way, we expect no burden or risks arising from feces sampling.

Saliva sampling

No burden or risk due to saliva sampling expected, as discussed for study B.

# INTRODUCTION AND RATIONALE

**Study A:**

Respiratory infection is one of the major causes of illness in infants. For example, RSV infects virtually every child before the age of 2 years. Each year, about 28,000 infants require medical care for RSV bronchiolitis in the Netherlands (Yu2013, Isaacs2013) of which approximately 2000 require hospitalisation with costs of €2000–€4000 per patient (Meijboom2012, Bos2007, Jansen2007). Within a recent meta-analysis, one of the 8 risk factors for RSV-induced acute lower respiratory infection in children was “no breastfeeding”, with odds ratio of 2.24 (95% CI 1.56-3.20) (Rietveld2004). This clearly indicates that currently, infant formula does not offer the same level of protection to RSV as breastfeeding does. In 2006–2012, only an estimated 25% of infants in the WHO European Region were exclusively breastfed for the first 6 months, which is far below the global recommendation. Even though the rate of early initiation of breastfeeding is high in some countries, exclusive breastfeeding rates drop rapidly between 4 and 6 months of age and are very low at 6 months, subscribing the clear need for improvement of infant formula. Previously conducted research shows that apart from antibodies, other components in human protects children form infectious disease. Particularly research in human oligosaccharide shows that this may also be an important component in human milk that modulates infectious disease (Ramani2018).

Additionally, oligosaccharides seem to modulate the immune system as well and even seem to have an effect on the development of allergies in children. Children that received an infant formula enriched with the scGOS/lcFOS oligosaccharide mixture developed fewer allergies and eczema during the first year of life, compared to children that received infant formulas without scGOS/lcFOS(Arslanoglu2012).

It has been postulated that HMOS mediate protection against infections and allergies through two (possibly three) mechanisms. HMOS are supposed to have direct pathogen inhibiting effects. Duska et al. showed that some HMOS have a direct RSV-inhibiting effect. (Duska-McEwen2014). In addition, the potential of both neutral (LnNt) and acidic HMOS to effectively inhibit infection by specific pathogens is shown (Duska-McEwen2014). Mechanisms by which these specific HMOs exert their anti-infective properties are by inhibiting virus binding to the host cells and/or blocking virus entry into the cell or blocking viral replication within the cell. In addition to direct blockage of viral infection by mimicking viral receptors, indirect microbial and immune modulation by HMOS have been hypothesized.

In addition, immune modulatory effects have been demonstrated for 2’FL, suggesting an additional function of specific oligosaccharides (Goldman2007, Koning2015, Erney2000). HMOS have been postulated to modulate TLR signaling on immune cells and induce regulatory responses (Kulinich2016, He2016). For instance, 2-FL modulates CD14 expression in human enterocytes, thereby attenuating LPS-induced inflammation in vitro (He2016)). Moreover, it has nicely been shown that the addition of human milk as well as human milk oligosaccharides interact directly with the immune system in particular with DCs, through DC-Sign (Koning2015)). Siglecs and related glycan-binding proteins are essential in immune responses and immune regulation (Shang2013), providing ways for improvement of DC development. DCs are key in the identification of pathogens and directing the immune response towards effective immunity, including T cell and B cell responses. Because TLRs and C-type lectins are vital in pathogen recognition and immune modulation (essential during development of the immune system in early life) it seems likely that via multiple direct and indirect pathways specific HMOS contribute to the development of a balanced immune system, hence protection towards infections. Therefore, we postulate that HMOS are involved in regulation of mucosal immune function and this will be investigated further in the present proposal.

In addition, the role of microbial modulation of specific HMOS structures have been subject of extensive studies. More specifically, Maternal fucosyltransferase 2 status has been suggested to affects the gut bifidobacterial communities of breastfed infants (Puccio2017). In addition, Utilization of major fucosylated and sialylated human milk oligosaccharides by isolated human gut microbes have been shown (Thurl2010). Its correlation towards respiratory infection susceptibility however remains to be established.

Aside from the HMOS and antibodies, other components may play an important role as well in early life immune development. That is why we aim to build up a biobank to analyse additional components in the future. Components that may be of interest are specific fatty acids and cytokines. For instance, presence of IL-2 and IL-13 in human milk are associated with a reduction of eczema in children (Munblit2017). Additionally, the presence of IL-13 in colostrum is associated with a lower incidence of food allergies (Munblit2017). Hernell et al. showed that addition of specific fats (retrieved from cow’s milk) to plant-based infant formulas reduced the amount of infections in children (Hernell2016).

The components that we will identify to have a protective effect, we will further analyse in our laboratories to unravel their underlying mechanism. Not only will this aid to improve infant formulas, but this will also lead to new therapeutic strategies to treat (respiratory tract) infections and allergies.

Combined, this collaboration will lead to new insights in the components of breastmilk (including macro and micro nutrients, proteins, peptides, lipids, amino-acids, but specifically HMOS) and the role they play in providing protection to infants. More knowledge regarding by which mechanism the specific HMOS structures can provide protection towards specific pathogens, will help to further improve infant formulas to prevent infections and allergies

**Study B:**

In the sub study we will also collect additional samples to research what the influence of breastfeeding is on immune development, compared to other routes that are enrolled in the transfer of maternal immunity to the neonate. This will be done in a subgroup of 20 mother-child dyads. The samples that we will collect are cord blood samples, a maternal blood sample and an amniotic fluid sample. Jacobino et al. showed that antibodies retrieved form human amniotic fluid protected mice pups against RSV-infections (Jacobino2016). Very little is known about the protective value antibody titters in cord blood. Active placental transport of maternal antibodies to the neonatal blood has been described, but little is known about the effectiveness of these antibodies (Kohler1966). To obtain more insight in the effect of the antibodies that are being transferred by breastfeeding, we will compare the effect of breastfeeding to the antibodies in cord blood and amniotic fluid. To evaluate the effect of the maternal Bordetella pertussis vaccination on (pertussis-specific) immune development in children, we will also collect saliva samples at 1 week, 1 month, 3 months, 6 months and 12 months postpartum. Saliva samples offer an ideal method to evaluate the (Bordetella-pertussis specific) immune development during the first year of life since saliva antibody titters correlate well with serum titters but saliva samples are considered to be non-invasive.

**Study C:**

In an additional sub-study (afterwards called study C) we would like to expand the current study design to assess the role of the maternal diet and infants’ intestinal microbial development on the risk of developing respiratory tract infections in the first year of life.

Breast milk is a very variable body fluid influenced by many maternal and environmental factors (Munblit et al., 2014). The maternal diet during lactation is one of the factors for which there are strong suggestions that it influences breast milk composition (Bravi et al., 2016). A Finnish observational cohort study with normal weight (n=49) and overweight (n=51) women found an association of a 34% percent increase of n-3 fatty acids (FA) in a 3 month postpartum-breast milk sample for women who consumed fatty fish in the two days before the sample collection. The same study found a significantly increased n-6 FA concentration in breast milk of women who consumed vegetable-oil-based-spreads on a regular basis. Consumption of high-fat dairy products was associated with a significantly increased concentration of short-chain fatty acids (SCFAs) (Mäkelä et al., 2013).

As elaborately discussed in the background for study A, breastfeeding protects against respiratory tract infections in infants, including RSV infection. Apart from antibodies and HMOs, also FAs have antiviral and -microbial properties. Especially medium chain saturated, such as lauric acid (C12:0), and long chain unsaturated FAs, such as linoleic acid (C18:2) appear to be potent in affecting the lipid coating of viruses (Isaacs, 2005; Thormar et al., 1987). In addition, macrophages exposed to omega-3 FA docohexaenoic acid (DHA) (C22:6) and omega-6 FA arachidonic acid (C20:4) have an increased capacity to phagocytise and have an increased microbicidal activity. Endogenous fatty acid production by the mammary gland is limited to a chain length of 14 (*de novo* FAs), therefore, the long chain FAs are derived from the diet (Hachey et al., 1989; Nasser et al., 2010), indicating the relevance of the maternal diet in relation to risk of RTIs for infants.

Very limited research has been performed focusing at the association between maternal diet and HMO composition in breast milk. A prospective observational birth cohort did not find a correlation between maternal diet quality and total HMO concentration nor for individual HMOs (after correction for multiple comparisons) in breast milk (Azad et al., 2018). However, the study of Azad and colleagues assessed the maternal diet by means of a food frequency questionnaire (FFQ) during late pregnancy, while the breast milk sample was collected 3-4 months postpartum. Another prospective cohort did find a positive correlation between fruit intake and unsaturated fatty acids consumed on the day before breast milk sampling, with various (sulfonated) HMOs in breast milk. Even more, monosaccharide 5-N-glycolyl-neuraminic acid (Neu5Gc) was identified in all breast milk samples. Notably, this monosaccharide cannot be synthesized endogenously and is diet-derived (e.g. from red meat) (Quin et al., 2020).

HMOs are of increasing interest because of their antimicrobial and antiviral capacity on one hand, and prebiotic capability on the other hand. HMOs prevent viruses to adhere to epithelial surfaces, by mimicking viral receptors or block receptors on epithelial cells (Morozov et al., 2018). Their prebiotic effect is achieved by the fact that specific strains of the commensal species Bifidobacteria and Bacteroides are able to metabolize HMOs and therefore are in favour in competition with strains unable to metabolize HMOs, such as *E. coli* and *Enterococci* (Ayechu-Muruzabal et al., 2018)*.*

Accumulating evidence shows that the intestinal microbial composition mediates the lung microbiota composition, also referred to as the gut-lung axis. The presence of microbiota in the respiratory tract, and more importantly the significant role it has in the pathogenesis of respiratory tract infections, is only studied for about a decade. Schuijt and colleagues performed a mice study for which mice were treated with antibiotics, after which *S. pneumonia* was administered intranasally. It was found that the antibiotic-treated mice had a.o. an increased mortality, inflammatory markers and an increased bacteria outgrowth. The significant role of the intestinal microbiota specifically was shown by normalizing the bacterial counts and IL-10 and TNF-α levels in the respiratory tract after fecal microbiome transplantion (FMT) (Schuijt et al., 2016). A different mice study of Kanmani and colleagues showed the relevance of the lung microbiota in the context of respiratory tract infections. Mice were intranasally administrated with *Corynebacterium pseudodiphteriticium,* a bacterium which is a commensal in the respiratory tract microbiota. The priming appeared to reduce the susceptibility to a primary RSV infection by modulating the toll-like receptor 3-mediated antiviral inflammatory response (Kanmani et al., 2017). The microbiota community present in the lung also seems to be relevant for the development of asthma. Comparison of the lung microbiota of both asthmatic adults and children shows that there was an increased presence of pathogenic Proteobacteria compared to the healthy controls (Hilty et al., 2010). Remot and colleagues performed a study with mice, for which they isolated several strains from the lung microbiota of healthy mice. The authors showed that intranasal administration of isolated strain to neonatal mice influenced the asthmatic immune response towards a house dust mite challenge (Remot et al., 2017).

Considering the influence of HMOs on the intestinal microbial development in infants, and the careful associations between intestinal microbial composition and risk of developing of RTIs and/or asthma, the pathway from maternal diet to breast milk composition to intestinal composition to eventually risk of developing RTIs and/or allergies is very relevant.

Taking together, although there are strong suggestions for the influence of maternal diet on breast milk composition, the scarcity of high quality studies and diversity in study design restrain the ability to draw strong conclusions. In addition, there is a lack of studies linking the maternal diet and associated breast milk composition to the gut health of the infant resulting in protection against respiratory infections and/or the development of allergies. There is a vast need for an observational study with a bigger sample size, which is also based on a power calculation (Bravi et al., 2016). Therefore, we plan to perform a substudy within the PRIMA human milk cohort. In this cohort (n=250), we will additionally assess the maternal diet, and collect feces and saliva of the infant. This study design allows to not only link the short- and long-term influence of maternal diet to breast milk composition, but also the clinical health outcomes in infants, with Medically Attended Respiratory tract Infections during the first year of life as main study parameter. Even more, the influence of the maternal diet

and an infant’s antibody repertoire on intestinal microbial composition, and subsequently on the risk of developing RTIs is explored.

# OBJECTIVES

**Study A:**

Primary objective:
To measure to what extent 2’FL human milk oligosaccharides are protective against medically attended respiratory tract infection during the first year of life.

Secondary objective 1:

To identify which components, or combination of components, in human milk are protective against any parent-reported, physician reported infections during the first year of life.

Secondary objective 2:

To identify which components, or combination of components, in human milk are protective against the development of parent-reported, physician reported allergies during the first year of life and.

Secondary objective 3:

To identify which immunological components (antibodies, immune cells) in human milk, cord blood and amniotic fluid are protective against any parent-reported, physician reported infections during the first year of life.

Secondary objective 4:

To obtain insight in the gut discomfort and crying of infants in the first 6 months of life.

**Study B:**

Secondary objective 5:

To describe antibody concentrations in compartments relevant to materno-fetal antibody transfer. We will define the association between maternal serum, breast milk antibodies, cord blood, amniotic and infant antibodies over time. This study is performed while maternal pertussis vaccination is introduced. We will therefore analyse total and pertussis-specific antibody concentrations.

**Study C:**

Secondary objective 6:

To identify the long-term and short-term influence of the maternal diet on the breast milk composition.

Secondary objective 7:

Link the long-term and short-term influence of the maternal diet to the clinical outcome of respiratory tract infection during the first year of life.

Secondary objective 8:

To derive hypotheses about potential mechanisms if the maternal diet-influenced components in human milk are protective against respiratory tract infection during the first year of life.

Secondary objective 9:

To identify the association between long-term and short-term influence of the maternal diet on the microbiota composition of a 3-month old infant, and its consequence for the risk of developing respiratory tract infections in the first year of life.

Secondary objective 10:

To identify the antibody repertoire and viral genome in saliva samples from 3 month-old infants.

Secondary objective 11:

To link the antibody repertoire in saliva samples with antibody repertoire in feces and microbiota composition, and how this relates to the risk of developing respiratory tract infections in the first year of life.

#
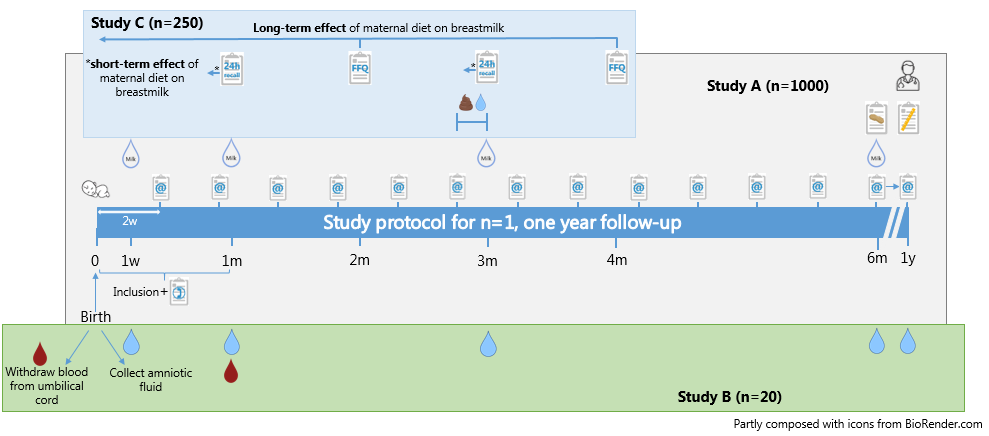
STUDY DESIGN

**Study A**

This cohort concerns a prospective observational cohort study. A retrospective collection of data collection or sample collection would not be useful for this cohort. Retrospective data collection will decrease the validity of data collected from parents through questionnaires. Moreover, since the composition of breast milk differs in time postpartum, samples have to be collected at certain time points in order to elucidate its precise and unique composition.

**Study B**

Saliva samples will be collected at 1 week, 1 month, 3 and 6 months because of pragmatic reasons. The 12 months sample will be collected since the last vaccination during the first year of life against Bordetella pertussis is offered at 11 months (the next vaccination is offered at 4 years in the Netherlands) (Rijksvaccinatieprogramma).

**Study C**

A 24h recall is filled in on the day of collection of the 1 and 3 month breast milk sample, to determine the short-term effect of maternal diet on breast milk composition. The FFQ is filled in at 2 and 4 months, to determine the long-term effect (dietary pattern) on breast milk composition. The 4 month FFQ is a repetitive measurement to evaluate whether the maternal diet has changed since 2 months, and therefore also can be linked to the 3 month breast milk sample. A change in dietary pattern is conceivable because, for example, the mother has stopped breastfeeding and/or maternity leave from work has ended.

Feces and saliva are collected at 3 months because at this time point the effect of the breast milk composition is expected to more significant that at 1 month or 1 week postpartum. The 3 month time point is also more suitable than the 6 month time point because by that time, infants may have received solid foods aside of breast milk and/or infant formula.

# STUDY POPULATION

## Population (base)

Participants will mainly be recruited from the obstetric wards at the Wilhelmina Children’s Hospital (WKZ) and Diakonessenhuis (Utrecht, the Netherlands).

As women who delivered without complications, may leave the hospital, we will approach these women who delivered at WKZ as well. This letter is added to the discharge letter/package in an envelope with a bright coloured label. The recruitment is further explained in paragraph 8.2. and depicted in the flowchart below:


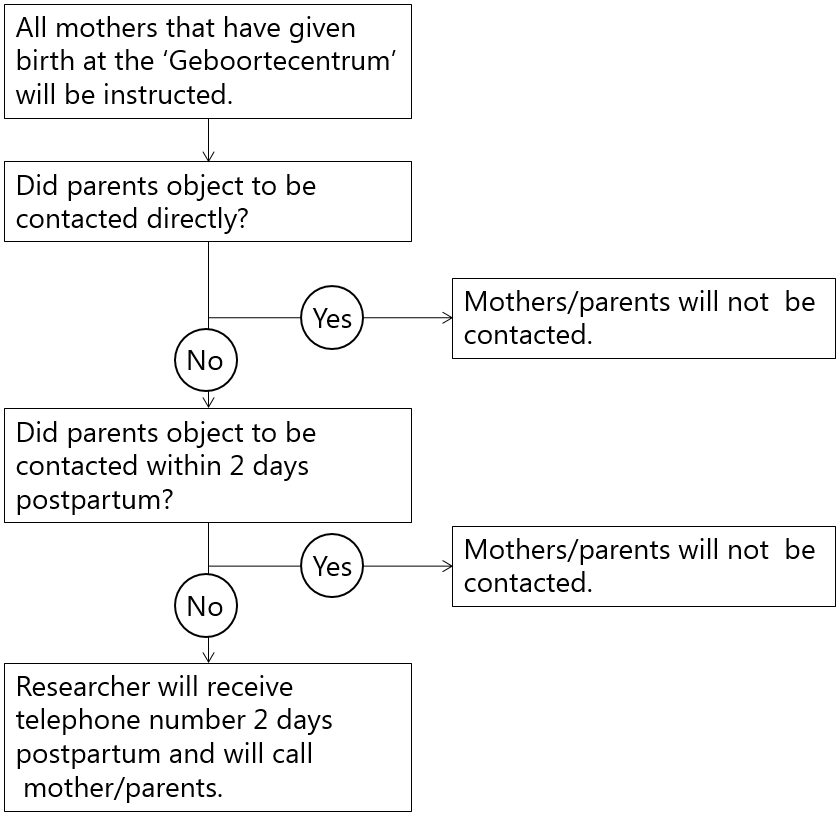


The researchers will report to METC Utrecht the amount of parents that were not properly informed about the research by healthcare workers after contacting the first 50 mothers.

At the time an initiation visit will be planned, we will already send the informed consent and Patient Information Letter via e-mail.

## Inclusion criteria

In order to be eligible to participate in this study, a subject must meet all of the following criteria:

- All mother-child dyads that live in a proximity to Utrecht (because of logistical issues) and/or gave birth at the WKZ or Diakonessenhuis.
- Parents should have the intention to continue breastfeeding until at least 3 months postpartum.

## Exclusion criteria

A potential subject who meets any of the following criteria will be excluded from participation in this study:

- Concerning the child:
  - (inborn) severe cardiac or pulmonary disorders or other severe organ diseases
  - Extreme prematurity (defined as GA<32 weeks).
- Concerning parents:
  - Acquired or innate immune deficiencies (excluding asthma, eczema and allergies).
  - The (lactating) mother receives medication or has a physical condition which forms a contraindication for breastfeeding.
  - Insufficient control of the Dutch language (i.e. at least B1 level).

## Sample size calculation

## Study A:

A sample size of 1000 participants participants has an 80% power to detect a ratio of the means of 0.91 in those with vs. those without a medically attended LRTI using a two-sided two-sample t-test, assuming that 10% of the cohort participants have a medically attended LRTI (N=100) and that the 2’FL HMO concentration in the population has a mean of 3g/L, a standard deviation of 1g/L, and a normal distribution (Donovan2016). The significance level (alpha) is 0.05. The sample size was calculated using Power Analysis for Sample Size (PASS) 2008.

**Study B: .**

Group sample sizes of 9 and 9 achieve 82% power to detect a difference of 88.3 between the null hypothesis that both group means are 100.7 and the alternative hypothesis that the mean of group 2 is 12.4 with estimated group standard deviations of 80.0 and 18.0 and with a significance level (alpha) of 0.05 using a two-sided two-sample t-test (Maertens2016).

**Study C:**

From the questionnaires that will be used "24h recall' has the largest number of variables at 216 variables. Although, the number of necessary examples depend on the data itself, considering algorithms such as support vector machines will be used for the classification, we need at least the same number of examples as variables. This is because we will try to solve a system of linear equations, and to avoid and ill-conditioned system we need to have 1:1 relationship between variables and samples (Smola and Schölkopf, 2004). In addition, as we will divide our analysis into training and testing, we need an extra 10% of samples for testing and considering a measurement error of 5% puts the total of samples at 246.1 ~= 247 (upper limit). Thus, we need at least 247 samples for the analysis of the dataset.

# METHODS

## Study parameters/endpoints

### Main study parameter/endpoint

- Parent-reported medically attended respiratory tract infection during the first year of life.

### Secondary study parameters/endpoints (if applicable)

**Study A:**

- Other infections during the first year of life (non-MARI respiratory tract infections but also gastro-intestinal infections).
- Allergies developed during first year of life.
- Gut discomfort of the infant during the first 6 months of life.
- Breast milk composition: antibody repertoire, oligosaccharides, immune cell repertoire (n=1000), extracellular vesicles (n=15).

**Study B:**

- Antibody repertoire in amniotic fluid, cord blood, maternal blood and infant’s saliva.
- Oligosaccharide, extracellular vesicles, antibody and immune cell repertoire in blood and amniotic fluid.

**Study C:**

- Diet(ary pattern) of the lactating mother.
- Fatty acid, protein, amino acid, fat- and water soluble vitamins and fibres composition in breast milk.
- Microbial composition of infant’s feces.
- Antibody repertoire and viral genome of infant’s saliva.

### Other study parameters (if applicable)

Other parameters that will be measured are:

- Complications and medication use during pregnancy and during labour.
- Birth characteristics (e.g. birth weight, Apgar score)
- Feeding habits (e.g. high allergenic food during pregnancy and first feeding of child)
- Household characteristics (e.g. age and amount of other children in household, pets etc.)
- Smoking habits of parents
- General information of parents (age, genetic background, education level, presence of (chronic) diseases and allergic status)
- Vaccination status mother

See for all questions the baseline and 2-weelky questionnaire.

## Study procedures

*Breast milk sampling:*

Women who participate in this study are asked to express and collect a whole milk sample from both breasts. The sample will be mixed by gently tilting the flask. After mixing, 5-10mL of the whole sample will be stored in a 50mL Falcon tube in the fridge. Parents are asked to note the date, time of expression and storage temperature on the tube. Samples will be handed in to the researcher during a house visit. The researcher will note the moment (1 week, 1, 3 or 6 month postpartum) and type (fore-, hind- or whole milk) of breast milk sample. The researcher will transport the sample to the UMC Utrecht. (Miller2013) The optimal processing conditions to fractionate the samples were tested in a pilot, see our SOP for more details about the processing of human milk samples before storage.

**Study C:**

For the breast milk sampling from mothers participating in study C, mothers are asked to collect a breast milk sample on the morning of collection before breakfast. This is asked because the assessment for the short-term influence of the maternal diet (24h recall) concerns the day before collection.

*Cord blood sampling:*

Cord blood is sampled from the umbilical vein, following the delivery of the child. First the umbilical has to be double clamped 3-5cm from the umbilicus to transect the umbilical cord. The child will consequently be removed from the field. The cord will now be prepared for blood sampling using a povidone iodine applicator to sterilize the umbilicus. Next, the needle will be inserted in the umbilical vein, standard blood sampling tubes will be used to collect the cord blood. The blood can be collected both *in utero* or *ex utero*. Meaning the venipuncture can be performed after or before the placental delivery. (Amson2015)

*Amniotic fluid collection:*

After rupturing the amniotic sac, amniotic fluid will be collected (regular procedure at the obstetric ward). The amniotic fluid will be stored at room temperature until retrieved by the researchers. For the study, about 200mL cord blood will be collected. 100ml will be used for cell analysis. About 9 vials with 1x10^7 cells each will be stored in the biobank, the other 1x10^7 cells will directly be used for FACS analysis. The remaining 100ml will be used to obtain serum. We will use about 1-2mL of serum to measure antibodies and HMOS, the remaining serum will be stored at the biobank facility of the UMC Utrecht.

*Venipuncture:*

Maternal blood samples will preferable be collected during the first house visit and not at delivery. We will collect venous blood samples from the median cubital vein.

We will analyse these blood samples on various components. The antibody composition will be performed in the group of Jeanette Leusen (LTI, UMC Utrecht) using ELISA or multiplex approaches. Oligosaccharide composition wil be measured in the group of Belinda van ’t Land (LTI UMC Utrecht and Nutricia). Immune cell repertoire will be analysed in the group of Jeanette Leusen and/or Femke van Wijk (LTI, UMC Utrecht) using flow cytometry. Extracellular vesicles will be measured at the group of Marca Wauben (Faculty of veterinary medicine, Utrecht University). We will collect about 70mL blood. We will collect five 10mL sodium-heparin tubes, of which one will be used for FACs analysis. The other tubes will be stored at the biobank. We will also collect two 10mL serum tubes. 1-2mL of the serum will be used for antibody and HMOS analysis, the remaining serum will be stored at the biobank facility of the UMC Utrecht.

*Saliva sampling:*

**Study B:**

Saliva samples will be collected during the first week postpartum, at 1, 3, 6 and 12 months postpartum. Whenever children are already spilling saliva, this can be collected directly with a saliva swab (i.e. a soft inert polymer swab that will take up the saliva). When children are not spilling saliva, the saliva swab can also be introduced gently into the mouth in a similar way as a pacifier. The child has to suck or chew on the saliva swab for several seconds before the swab will be retrieved. The saliva swab will afterwards be stored in a collection tube on ice for transportation to the lab. At the lab, saliva will be removed for the swab by using a centrifuge. The collected saliva will be aliquoted in several tubes, maximal 900uL per tube. In case of lower amounts of saliva yields, tubes will contain less the 900uL, this will be logged for future sample usage.

**Study C:**

Saliva will be collected by a parent in the week before the 3 month sample collection. Saliva will be sampled with Micro•SAL™ for Children according to manufacturer’s protocol. The pad of the Collector will be placed in the mouth of the infant until the pad is saturated (60-90 sec.). The saturated collector will be pushed into the compression tube, which is firmly connected with its base into an Eppendorf tube. The Eppendorf tube with saliva sample will be stored at -20°C in the freezer at the participant’s home, until collection by the researcher.

*Questionnaires:*

**Study A:**

Data will be collected using questionnaires and by interviewing general practioners and/or pharmacists.

Questionnaires will be send to parents using Castor EDC. Parents will receive a baseline questionnaire at moment of entering the study, which will cost them about 20-30 minutes to fill in. Consecutively they will receive every 2 weeks a short questionnaire to monitor infectious episodes, feeding habits, gut discomfort and crying of the infant and to screen for any infectious episodes in mothers, which takes 1-2 minutes each to fill in. The 2 weekly questionnaire will be expended to a longer list at 6 months and 12 months postpartum to screen children for allergies developed during the first year. Parents will receive questionnaires through email.

**Study C:**

The questionnaires for the dietary assessment will be send via e-mail. Parents receive login information for the tool which is reached via a website page. The FFQ will be send at 2 month and 4 month postpartum and will take 45-60 min. to fill in each. The 24h recall will be send at 1 month and 3 month postpartum and will take 20-30 min. fill in each.

## Withdrawal of individual subjects

Subjects can leave the study at any time for any reason if they wish to do so without any consequences. The investigator can decide to withdraw a subject from the study for urgent medical reasons.

### Specific criteria for withdrawal (if applicable)

Only when not one breast milk sample is collected that is stored on the proper conditions (i.e. stored in the fridge or on ice after collection), participants can be withdrawn from the study

## Replacement of individual subjects after withdrawal

A new mother-child dyad will be recruited to replace a withdrawn dyads when withdraw occurs before the first sample is taken.

## Follow-up of subjects withdrawn from treatment

na.

## Premature termination of the study

na.

# SAFETY REPORTING

na

# STATISTICAL ANALYSIS

The section on statistical analysis only describes how the quantitative analyses will be done, answering the primary and secondary research objectives.

## Primary study parameter(s)

## The association between 2’FL concentrations and the number of medically attended respiratory tract infections during the first year of life will be determined using Poisson regression analysis, adjusted for potential confounders. Potential confounders will be identified by a literature search, complemented by expert knowledge. One such potential confounder is like 3’SL that is both suggested to exert a direct antiviral effect as well as to be associated with a shorter lactation duration (Azad201,; Laucirica2017, Pandey2018).

## We will also explore whether the moment of lactation postpartum, season and ethnicity modify the effect of 2’FL on the number of respiratory tract infections. Additionally we will search for combinations of components that have a protective effect, by incorporating combinations together with their interactions (e.g. seasonality, ethnicity of mother and time postpartum that sample is collected).

## Secondary study parameter(s)

## The association between 2’FL concentrations and the number of allergies and total number of infections during first year of life will be analysed in a similar fashion as for the primary outcomes.

The 24h recalls filled in by the mother, will be checked on missing data in the same week by trained staff When missing data is expected, for example 1 slice of bread but 5 portions of bread toppings are noted, the researcher or research nurse will contact the participant. Notes from the participants will also be processed by the trained staff.

## Other study parameters

See 10.2. and 10.3.

## Missing data

In case there is lost to follow-up because parents stop to report the questionnaires, we will use the data retrieved from the general practioner to estimate the amount of medically attended respiratory tract infections. If this is not possible for the amount of respiratory tract infections, we will impute the missing data.

# ETHICAL CONSIDERATIONS

## Regulation statement

The study will be conducted according to the principles of the Declaration of Helsinki (version, date, see for the most recent version: www.wma.net) and in accordance with the Medical Research Involving Human Subjects Act (WMO) and the International Code of Marketing of Breast milk Substitutes.

## Recruitment and consent

There are three ways by which the initial contact is made:

1. (research) nurses, researchers or midwives of the WKZ or Diakonessenhuis have direct contact with possible participants. This can be on the obstetric ward of WKZ and Diakonessenhuis (researcher, nurses, midwives or other healthcare workers working at the ward) or at home (during a visit of Kraambed-Nazorg, specific midwives that pay house visits after women gave birth at the WKZ). (research) nurses and midwives will provide information and ask whether they may be contacted by the research team. If so, the (research) nurses and midwives share contact information with the research team.
2. All women that will give labour at the UMC Utrecht will receive a letter at discharge from the hospital, to inform them that they will be contacted by researchers of the PRIMA study. This letter is added to the discharge letter/package in a bright coloured envelope. The following short narrative is added to the discharge package for the healthcare worker who will hand over this package:“ “In de komende dagen zouden onderzoekers u graag willen bellen of u wilt deelnemen aan een belangrijk onderzoek over borstvoeding. Vindt u dat goed? Zij zullen u inhoudelijk verder informeren. **Als u niet wilt worden benaderd kunt u dat nu aangeven, of binnen 48 uur via email of telefoon.** Graag de voor- en achternaam van uw kind en/of de moeder van het kind hierbij vermelden. Het e-mailadres en telefoonnummer vindt u in de envelop.”
   English translation: “In the following days researcher would like to call you about an important study on breastfeeding. They will further inform you about the study. **If you prefer not to be contacted about this study, please indicate this now or within 48 hours via email or by telephone**. Please mention the first and family name of your child and/or the mother of the child. The email address and telephone number can be found in the orange envelope.”
   As soon as parents directly have objections to be contacted, the healthcare worker will inform the researcher by email or telephone.
   Whenever no objection is registered, the researchers will receive the telephone number of these mothers after 2 days postpartum. The researchers will inform parents about the procedure how and why their telephone numbers were retrieved during the first phone call.
   Additionally, the researchers will register the amount of parents that indicate (not) to be informed. The researchers will use this as feedback to the healthcare workers at the obstetric ward.
   The researchers will report to the METC Utrecht the amount of parents that were not properly informed about the research by healthcare workers after contacting the first 50 mothers.
3. Advertisement at the UMC Utrecht corporate website.

If parents indicate interest in the trial, additional information will be send through email (i.e. the research folder, IC and patient information folder will be sent). If necessary, an additional phone call will be planned to give parents enough time to decide whether they would like to participate. If parents indicate they would like to participate, a first visit will be planned. First visits occur within 24 hours after the first contact and within the first week postpartum. The baseline questionnaire will be filled in during the initiation visit.

During the initiation visit, parents will have time to ask questions (if not done earlier) and the informed consent will be signed. Directly afterwards, human milk samples will be collected.

## Objection by minors or incapacitated subjects (if applicable)

This is not applicable to our study, since the only samples that will be collected from children, will be cord blood. Also the parents will fill in the questionnaires, not the child.

## Benefits and risks assessment, group relatedness

Benefits: No benefits are to be expected from participating in this trial.

Risk for children:

Risk for the children is supposed to be minimal. Only a data breach forms a possible risk to them, but this will be adequately avoided using our Data Management Plan (see for more information the Data Management Plan). There is no risk for the child associated to the collection of neonatal blood from the placenta after birth or with saliva sample collection. We assess that these risk are minimal, and since we will reduce this risk by our DMP, we deem this risks as acceptable.

Risk for mothers:

We also estimate that the risk for participating mothers is minimal. Risks associated with expressing milk are: pain during expression and concerns about milk production. Pain during expressing milk is mainly attributable to wrong techniques or a non-fitting pumping device. This can be overcome by using a properly fitting pump or by manual expression of milk. Also a data breach forms a possible risk to them, but this will be adequately avoided using our Data Management Plan (see for more information the Data Management Plan). There is a minimal risk associated with the venipuncture. Although considered safe, rarely phlebitis, extravasation of blood, bruising and hematoma forming following venipuncture have been reported. However, since complications form venipunctures and expressing milk are rare, and we will decrease the risk of a data breach as best as we can with our DMP, we asses these risks for mothers acceptable. No risk is associated with the collection of amniotic fluid.

## Compensation for injury

Since no therapeutic intervention is investigated and there is a minimal risk for participants, we request dispensation for a liability insurance.

## Incentives (if applicable)

Participants will not receive any special incentives, compensation or treatment for participation in the study.

# ADMINISTRATIVE ASPECTS, MONITORING AND PUBLICATION

## Handling and storage of data and documents

Data, samples and documents will be stored using a specific code. This code for samples consists of: study ID (i.e. UMCUPRIMA) an anonymised participant number (e.g. for participant 1 this will be 10001), moment of sampling (e.g. week 1 is W1) and sample number including type of sample (W for water fraction, F for fat and C for cellular fraction). Combining this codes will result in the following; for example participant 1 donated milk during the first 1 week postpartum and we will label a tube with the first part of the water fraction, the code will be: UMCUPRIMA.10001.W1.W01.

The coding system for neonatal blood samples from the placenta will be composed as follows: it consists of study ID (i.e. UMCUPRIMA) an anonymised participant number (e.g. for participant 1 this will be 10001) (this number will be similar to the milk sample number), indication it is neonatal blood (BLOOD.NEO)In case a mother gives labour to a twin, the N will be followed by either a 1 or 2. The 1 and 2 should correspond to the castor ID’s that will be used for sending the questionnaires.

The coding system for maternal blood samples will be composed as follows: it consists of study ID (i.e. UMCUPRIMA) an anonymised participant number (e.g. for participant 1 this will be 10001)(this number will be similar to the milk sample number), indication it is maternal blood (BLOOD.MAT).

The coding system for amniotic fluid samples will be composed as follows: it consists of study ID (i.e. UMCUPRIMA) an anonymised participant number (e.g. for participant 1 this will be 10001)(this number will be similar to the milk sample number), indication it is maternal blood (AMNIOTICF.).

The coding system for infant saliva samples will be composed as follows: it consists of study ID (i.e. UMCUPRIMA) an anonymised participant number (e.g. for participant 1 this will be 10001), indication infant saliva sample (SALIV.INF.), moment of sampling (e.g. week 1 is W1) and number of tube (e.g. the first tube is T1). Combining this codes will result in the following: for example the first tube of saliva sample of participant 1 that donated saliva during the first 1 week postpartum, the code will be: UMCUPRIMA.10001.SALIV.INF.W1.T1.

The coding system for infant feces samples will be composed as follows: it consists of the study ID (i.e. UMCUPRIMA), an anonymised participant number (e.g. for participant 1 this will be 10001), indication infant feces sample (FECES.INF.), moment of sampling (e.g. week 1 is W1) and number of tube (e.g. the first tube is T1). Combining this codes will result in the following for the example of the first tube of saliva sample of participant 1 that donated saliva 3 month postpartum, the code will be: UMCUPRIMA.10001.FECES.INF.M3.T1

The codes being used in Castor EDC will be numerical and will be generated by castor itself.

The key for the code(s) will be safeguarded by the Data manager. Only the data manager(s) have access to the key at any time. Researchers that work with personal data only have limited access to this key. Personal data researchers need to be able to contact participants for follow up samples and for house visits. It may happen that a researcher that currently is performing an analysis on the data, simultaneously need access to the personal data as well. In this case, the research data will maximally be pseudonomised as possible by an independent data manager to reduce the risk that these data can be traced back to personal data.

The key(s) will be stored digitally in a folder from the research Folder Structure that only is accessible for datamanager(s). This will be stored in a Research Folder Structure according to the protocol on Connect (version 1.2. January 20, 2019) Multiple datamanager(s) have access to the key(s) in order to avoid loss of the key.

For additional information about data storage, protection and safety, we refer to the data Management Plan. The data and human material will be stored for unlimited time.

## Monitoring and Quality Assurance

Monitoring of the study will take place at three time point: at initiation of the study, one midterm monitor visit and one close-out monitor visit. See for more details document ‘K6 Monitoringplan’.

## Amendments

Amendments are changes made to the research after a favourable opinion by the accredited METC has been given.

All substantial amendments will be notified to the METC and to the competent authority.

Non-substantial amendments will not be notified to the accredited METC and the competent authority, but will be recorded and filed by the sponsor.

## Annual progress report

The sponsor/investigator will submit a summary of the progress of the trial to the accredited METC once a year. Information will be provided on the date of inclusion of the first subject, numbers of subjects included and numbers of subjects that have completed the trial, serious adverse events/ serious adverse reactions, other problems, and amendments.

## Temporary halt and (prematurely) end of study report

The investigator/sponsor will notify the accredited METC of the end of the study within a period of 8 weeks. The end of the study is defined as the last patient’s last visit.

The sponsor will notify the METC immediately of a temporary halt of the study, including the reason of such an action.

In case the study is ended prematurely, the sponsor will notify the accredited METC within 15 days, including the reasons for the premature termination.

Within one year after the end of the study, the investigator/sponsor will submit a final study report with the results of the study, including any publications/abstracts of the study, to the accredited METC.

## Public disclosure and publication policy

All data arising from this study will be published. However, for patenting some components found to have a protective effect, Nutricia Research bv. reserved the right to delay publication for 3 months maximum.

# REFERENCES

1. Abu Raya B, Edwards KM, Scheifele DW, Halperin SA. Pertussis and influenza immunisation during pregnancy: a landscape review. Lancet Infect Dis. 2017 Jul;17(7):e209-e222.
2. Ajetunmobi OM, Whyte B, Chalmers J, Tappin DM, Wolfson L, Fleming M, MacDonald A, Wood R, Stockton DL. Breastfeeding is associated with reduced childhood hospitalization: evidence from a Scottish Birth Cohort (1997-2009). J Pediatr. 2015 Mar;166(3):620-5.
3. Amirthalingam G, Campbell H, Ribeiro S, Fry NK, Ramsay M, Miller E, Andrews N. Sustained Effectiveness of the Maternal Pertussis Immunization Program in England 3 Years Following Introduction. Clin Infect Dis. 2016 Dec 1;63(suppl 4):S236-S243.
4. Armson BA, Allan DS, Casper RF. Umbilical Cord Blood: Counselling, Collection, and Banking. J Obstet Gynaecol Can. 2015 Sep;37(9):832-844.
5. Arslanoglu S, Moro GE, Boehm G, Wienz F, Stahl B, Bertino E. Early neutral prebiotic oligosaccharide supplementation reduces the incidence of some allergic manifestations in the first 5 years of life. J Biol Regul Homeost Agents. 2012 Jul-Sep;26(3 Suppl):49-59.
6. Azad MB, Robertson B, Atakora F, Becker AB, Subbarao P, Moraes TJ, Mandhane PJ, Turvey SE, Lefebvre DL, Sears MR, Bode L. Human Milk Oligosaccharide Concentrations Are Associated with Multiple Fixed and Modifiable Maternal Characteristics, Environmental Factors, and Feeding Practices. J Nutr. 2018 Nov 1;148(11):1733-1742.
7. Blanken MO, Frederix GW, Nibbelke EE, Koffijberg H, Sanders EAM, Rovers MM4, Bont L. Cost-effectiveness of rule-based immunoprophylaxis against respiratory syncytial virus infections in preterm infants. Eur J Pediatr. 2018 Jan;177(1):133-144.
8. Bos JM, Rietveld E, Moll HA, Steyerberg EW, Luytjes W, Wilschut JC, de Groot R, Postma MJ. The use of health economics to guide drug development decisions: Determining optimal values for an RSV-vaccine in a model-based scenario-analytic approach. Vaccine. 2007 Sep 28;25(39-40):6922-9.
9. Caballero-Flores G, Sakamoto K, Zeng MY, Wang Y, Hakim J, Matus-Acuña V, Inohara N, Núñez G. Maternal Immunization Confers Protection to the Offspring against an Attaching and Effacing Pathogen through Delivery of IgG in Breast Milk. Cell Host Microbe. 2019 Feb 13;25(2):313-323.e4.
10. Donovan SM, Comstock SS. Human Milk Oligosaccharides Influence Neonatal Mucosal and Systemic Immunity. Ann Nutr Metab. 2016;69 Suppl 2:42-51.
11. Duijts L, Jaddoe VW, Hofman A, Moll HA. Prolonged and exclusive breastfeeding reduces the risk of infectious diseases in infancy. Pediatrics. 2010 Jul;126(1):18-25.
12. Duijts L, Jaddoe VW, Hofman A, Moll HA. Prolonged and exclusive breastfeeding reduces the risk of infectious diseases in infancy. Pediatrics. 2010 Jul;126(1):18-25.
13. Duska-McEwen G, Senft AP, Ruetschilling, TL, Barrett EG, Buck, RH. Human Milk Oligosaccharides Enhance Innate Immunity to Respiratory Syncytial Virus and Influenza in Vitro. Food and Nutrition Sciences. 2014 Jul; 5: 20141387-1398.
14. Erney RM, Malone WT, Skelding MB, Marcon AA, Kleman-Leyer KM, O'Ryan ML, Ruiz-Palacios G, Hilty MD, Pickering LK, Prieto PA. Variability of human milk neutral oligosaccharides in a diverse population. J Pediatr Gastroenterol Nutr. 2000 Feb;30(2):181-92.
15. Gaillard ME, Bottero D, Zurita ME, Carriquiriborde F, Martin Aispuro P, Bartel E, Sabater-Martínez D, Bravo MS, Castuma C, Hozbor DF. Pertussis Maternal Immunization: Narrowing the Knowledge Gaps on the Duration of Transferred Protective Immunity and on Vaccination Frequency. Front Immunol. 2017 Sep 6;8:1099.
16. Goldman AS. The immune system in human milk and the developing infant. Breastfeed Med. 2007 Dec;2(4):195-204.
17. He Y, Lawlor NT, Newburg DS. Human Milk Components Modulate Toll-Like Receptor-Mediated Inflammation. Adv Nutr. 2016 Jan 15;7(1):102-11.
18. Heath PT, Culley FJ, Jones CE, Kampmann B, Le Doare K, Nunes MC, Sadarangani M, Chaudhry Z, Baker CJ, Openshaw PJM. Group B streptococcus and respiratory syncytial virus immunisation during pregnancy: a landscape analysis. Lancet Infect Dis. 2017 Jul;17(7):e223-e234.
19. Hernell O, Timby N, Domellöf M, Lönnerdal B. Clinical Benefits of Milk Fat Globule Membranes for Infants and Children. Pediatr. 2016 Jun;173 Suppl:S60-5.
20. Hettegger P, Huber J, Paßecker K, Soldo R, Kegler U, Nöhammer C, Weinhäusel A. High similarity of IgG antibody profiles in blood and saliva opens opportunities for saliva based serology. PLoS One. 2019 Jun 20;14(6):e0218456.
21. Hirschmugl B, Brandl W, Csapo B, van Poppel M, Köfeler H, Desoye G, Wadsack C, Jantscher-Krenn E. Evidence of Human Milk Oligosaccharides in Cord Blood and Maternal-to-Fetal Transport across the Placenta. Nutrients. 2019 Nov 4;11(11).
22. Isaacs D. Should respiratory care in preterm infants include prophylaxis against respiratory syncytial virus? The case against. Paediatr Respir Rev. 2013 Jun;14(2):128-9.
23. Jacobino SR, Nederend M, Hennus M, Houben ML, Ngwuta JO, Viveen M, Coenjaerts FEJ, Hack CE, van Neerven RJJ, Graham BS, Bont L, Leusen JHW. Human amniotic fluid antibodies protect the neonate against respiratory syncytial virus infection. J Allergy Clin Immunol. 2016 Nov;138(5):1477-1480.e5.
24. Jansen AG, Sanders EA, Hoes AW, van Loon AM, Hak E. Influenza- and respiratory syncytial virus-associated mortality and hospitalisations. Eur Respir J. 2007 Dec;30(6):1158-66.
25. Koning N, Kessen SF, Van Der Voorn JP, Appelmelk BJ, Jeurink PV, Knippels LM, Garssen J, Van Kooyk Y. Human Milk Blocks DC-SIGN-Pathogen Interaction via MUC1. Front Immunol. 2015 Mar 13;6:112.
26. Kulinich A, Liu L. Human milk oligosaccharides: The role in the fine-tuning of innate immune responses. Carbohydr Res. 2016 Sep 2;432:62-70.
27. Laucirica DR, Triantis V, Schoemaker R, Estes MK, Ramani S. Milk Oligosaccharides Inhibit Human Rotavirus Infectivity in MA104 Cells. J Nutr. 2017 Sep;147(9):1709-1714.
28. Litt DJ, Samuel D, Duncan J, Harnden A, George RC, Harrison TG. Detection of anti-pertussis toxin IgG in oral fluids for use in diagnosis and surveillance of Bordetella pertussis infection in children and young adults. J Med Microbiol. 2006 Sep;55(Pt 9):1223-8.
29. Maertens K, Caboré RN, Huygen K, Hens N, Van Damme P, Leuridan E. Pertussis vaccination during pregnancy in Belgium: Results of a prospective controlled cohort study. Vaccine. 2016 Jan 2;34(1):142-50.Marchant A, Sadarangani M, Garand M, Dauby N, Verhasselt V, Pereira L, Bjornson G, Jones CE, Halperin SA, Edwards KM, Heath P, Openshaw PJ, Scheifele DW, Kollmann TR. Maternal immunisation: collaborating with mother nature. Lancet Infect Dis. 2017 Jul;17(7):e197-e208.
30. Meijboom MJ, Rozenbaum MH, Benedictus A, Luytjes W, Kneyber MC, Wilschut JC, Hak E, Postma MJ. Cost-effectiveness of potential infant vaccination against respiratory syncytial virus infection in The Netherlands. Vaccine. 2012 Jun 29;30(31):4691-700.
31. Miller EM, Aiello MO, Fujita M, Hinde K, Milligan L, Quinn EA. Field and laboratory methods in human milk research. Am J Hum Biol. 2013 Jan-Feb;25(1):1-11.
32. Morrow AL, Meinzen-Derr J, Huang P, Schibler KR, Cahill T, Keddache M, Kallapur SG, Newburg DS, Tabangin M, Warner BB, Jiang X. Fucosyltransferase 2 non-secretor and low secretor status predicts severe outcomes in premature infants. J Pediatr. 2011 May;158(5):745-51.
33. Munblit D, Treneva M, Peroni DG, Colicino S, Chow LY, Dissanayeke S, Pampura A, Boner AL, Geddes DT, Boyle RJ, Warner JO. Immune Components in Human Milk Are Associated with Early Infant Immunological Health Outcomes: A Prospective Three-Country Analysis. Nutrients. 2017 Jun; 9(6): 532.
34. Pandey RP, Kim DH, Woo J, Song J, Jang SH, Kim JB, Cheong KM, Oh JS, Sohng JK. Broad-spectrum neutralization of avian influenza viruses by sialylated human milk oligosaccharides: in vivo assessment of 3'-sialyllactose against H9N2 in chickens. Sci Rep. 2018 Feb 7;8(1):2563.
35. Pou C, Nkulikiyimfura D, Henckel E, Olin A, Lakshmikanth T, Mikes J, Wang J, Chen Y, Bernhardsson AK, Gustafsson A, Bohlin K2,3, Brodin P. The repertoire of maternal anti-viral antibodies in human newborns. Nat Med. 2019 Apr;25(4):591-596. doi: 10.1038/s41591-019-0392-8.
36. Puccio G, Alliet P, Cajozzo C, Janssens E, Corsello G, Sprenger N, Wernimont S, Egli D, Gosoniu L, Steenhout P. Effects of Infant Formula With Human Milk Oligosaccharides on Growth and Morbidity: A Randomized Multicenter Trial. J Pediatr Gastroenterol Nutr. 2017 Apr;64(4):624-631.
37. Ramani S, Stewart CJ, Laucirica DR, Ajami NJ, Robertson B, Autran CA, Shinge D, Rani S, Anandan S, Hu L, Ferreon JC, Kuruvilla KA, Petrosino JF, Venkataram Prasad BV, Bode L, Kang G, Estes MK. Human milk oligosaccharides, milk microbiome and infant gut microbiome modulate neonatal rotavirus infection.Nat Commun. 2018 Nov 27;9(1):5010.
38. Rietveld E, De Jonge HC, Polder JJ, Vergouwe Y, Veeze HJ, Moll HA, Steyerberg EW. Anticipated costs of hospitalization for respiratory syncytial virus infection in young children at risk. Pediatr Infect Dis J. 2004 Jun;23(6):523-9.
39. Rijksoverheid. www.rijksoverheid.nl; 2018. Blokhuis kondigt grootschalige extra vaccinatie aan: Ruim half miljoen kinderen krijgen oproep [updated 17 Jul 2018; cited at 16 Nov 2019]; [about 3 screens]. Available from: <https://www.rijksoverheid.nl/ministeries/ministerie-van-volksgezondheid-welzijn-en-sport/nieuws/2018/07/17/blokhuis-kondigt-grootschalige-extra-vaccinatie-aan-ruim-half-miljoen-kinderen-krijgen-oproep>
40. Rijksvaccinatieprogramma. https://rijksvaccinatieprogramma.nl/. Vaccinatieschema [updated 12 Jun 2019; cited at 16 Nov 2019];]; [about 3 screens]. Available from: https://rijksvaccinatieprogramma.nl/vaccinaties/vaccinatieschema
41. Shang J, Piskarev VE, Xia M, Huang P, Jiang X, Likhosherstov LM, Novikova OS, Newburg DS, Ratner DM. Identifying human milk glycans that inhibit norovirus binding using surface plasmon resonance. Glycobiology. 2013 Dec;23(12):1491-8.
42. Shi T, Balsells E, Wastnedge E, Singleton R, Rasmussen ZA, Zar HJ, Rath BA, Madhi SA, Campbell S, Vaccari LC, Bulkow LR, Thomas ED, Barnett W, Hoppe C, Campbell H, Nair H. Risk factors for respiratory syncytial virus associated with acute lower respiratory infection in children under five years: Systematic review and meta-analysis. J Glob Health. 2015 Dec;5(2):020416.
43. Thurl S, Munzert M, Boehm G, Matthews C, Stahl B. Systematic review of the concentrations of oligosaccharides in human milk. Nutr Rev. 2017 Nov 1;75(11):920-933.
44. Walker WA, Iyengar RS. Breast milk, microbiota, and intestinal immune homeostasis. Pediatr Res. 2015 Jan;77(1-2):220-8.
45. Xiao L, Leusink-Muis T, Kettelarij N, van Ark I, Blijenberg B, Hesen NA, Stahl B, Overbeek SA, Garssen J, Folkerts G, van’t Land B. human Milk Oligosaccharide 2′-Fucosyllactose improves innate and adaptive immunity in an in uenza-speci c Murine Vaccination Model. Front Immunol. 2018 Mar; 9:452.
46. Yu ZT, Chen C, Newburg DS. Utilization of major fucosylated and sialylated human milk oligosaccharides by isolated human gut microbes. Glycobiology. 2013 Nov;23(11):1281-92.
47. Ayechu-Muruzabal, V., van Stigt, A. H., Mank, M., Willemsen, L. E. M., Stahl, B., Garssen, J., & van’t Land, B. (2018). Diversity of human milk oligosaccharides and effects on early life immune development. Frontiers in Pediatrics, 6(September), 1–9. https://doi.org/10.3389/fped.2018.00239
48. Azad, M. B., Robertson, B., Atakora, F., Becker, A. B., Subbarao, P., Moraes, T. J., Mandhane, P. J., Turvey, S. E., Lefebvre, D. L., Sears, M. R., & Bode, L. (2018). Human Milk Oligosaccharide Concentrations Are Associated with Multiple Fixed and Modifiable Maternal Characteristics, Environmental Factors, and Feeding Practices. Journal of Nutrition, 148(11), 1733–1742. https://doi.org/10.1093/jn/nxy175
49. Bravi, F., Wiens, F., Decarli, A., Dal Pont, A., Agostoni, C., & Ferraroni, M. (2016). Impact of maternal nutrition on breast-milk composition: A systematic review. American Journal of Clinical Nutrition, 104(3), 646–662. https://doi.org/10.3945/ajcn.115.120881
50. Hachey, D. L., Silber, G. H., Wong, W. W., & Garza, C. (1989). Human lactation II: Endogenous fatty acid synthesis by the mammary gland. Pediatric Research, 25(1), 63–68. https://doi.org/10.1203/00006450-198901000-00015
51. Hilty, M., Burke, C., Pedro, H., Cardenas, P., Bush, A., Bossley, C., Davies, J., Ervine, A., Poulter, L., Pachter, L., Moffatt, M. F., & Cookson, W. O. C. (2010). Disordered microbial communities in asthmatic airways. PLoS ONE, 5(1). https://doi.org/10.1371/journal.pone.0008578
52. Isaacs, C. E. (2005). Human milk inactivates pathogens individually, additively, and synergistically. Journal of Nutrition, 135(5), 1286–1288. https://doi.org/10.1093/jn/135.5.1286
53. Kanmani, P., Clua, P., Vizoso-Pinto, M. G., Rodriguez, C., Alvarez, S., Melnikov, V., Takahashi, H., Kitazawa, H., & Villena, J. (2017). Respiratory commensal bacteria Corynebacterium pseudodiphtheriticum improves resistance of infant mice to respiratory syncytial virus and Streptococcus pneumoniae superinfection. Frontiers in Microbiology, 8(AUG), 1–14. https://doi.org/10.3389/fmicb.2017.01613
54. Mäkelä, J., Linderborg, K., Niinikoski, H., Yang, B., & Lagström, H. (2013). Breast milk fatty acid composition differs between overweight and normal weight women : the STEPS Study Index of Diet Quality. 727–735. https://doi.org/10.1007/s00394-012-0378-5
55. Morozov, V., Hansman, G., Hanisch, F. G., Schroten, H., & Kunz, C. (2018). Human Milk Oligosaccharides as Promising Antivirals. Molecular Nutrition and Food Research, 62(6), 1–14. https://doi.org/10.1002/mnfr.201700679
56. Munblit, D., Boyle, R. J., Warner, J. O., Warner, J. O., & Fleming, W. (2014). Factors affecting breast milk composition and potential consequences for development of the allergic phenotype Experimental Allergy. 583–601. https://doi.org/10.1111/cea.12381
57. Nasser, R., Stephen, A. M., Goh, Y. K., & Clandinin, M. T. (2010). The effect of a controlled manipulation of maternal dietary fat intake on medium and long chain fatty acids in human breast milk in Saskatoon, Canada. International Breastfeeding Journal, 5, 13–15. https://doi.org/10.1186/1746-4358-5-3
58. Quin, C., Vicaretti, S. D., Mohtarudin, N. A., Garner, A. M., Vollman, D. M., Gibson, D. L., Zandberg, W. F., & Hart, G. W. (2020). Influence of sulfonated and diet-derived human milk oligosaccharides on the infant microbiome and immune markers. Journal of Biological Chemistry, 295(12), 4035–4048. https://doi.org/10.1074/jbc.RA119.011351
59. Remot, A., Descamps, D., Noordine, M. L., Boukadiri, A., Mathieu, E., Robert, V., Riffault, S., Lambrecht, B., Langella, P., Hammad, H., & Thomas, M. (2017). Bacteria isolated from lung modulate asthma susceptibility in mice. ISME Journal, 11(5), 1061–1074. https://doi.org/10.1038/ismej.2016.181
60. Thormar, H., Isaacs, C. E., Brown, H. R., Barshatzky, M. R., & Pessolano, T. (1987). Inactivation of enveloped viruses and killing of cells by fatty acids and monoglycerides. Antimicrobial Agents and Chemotherapy, 31(1), 27–31. https://doi.org/10.1128/AAC.31.1.27
61. Smola, Alex J., and Bernhard Schölkopf. "A tutorial on support vector regression." Statistics and computing 14.3 (2004): 199-222.
